# Exploring the influence of cytosolic and membrane FAK activation on YAP/TAZ nuclear translocation

**DOI:** 10.1016/j.bpj.2021.09.009

**Published:** 2021-09-10

**Authors:** Kerbaï Saïd Eroumé, Rachel Cavill, Katerina Staňková, Jan de Boer, Aurélie Carlier

**Affiliations:** 1 MERLN Institute for Technology-Inspired Regenerative Medicine, Maastricht University, Maastricht, the Netherlands; 2 Department of Data Science and Knowledge Engineering, Faculty of Science and Engineering, Maastricht University, Maastricht, the Netherlands; 3 Department of Biomedical Engineering and Institute for Complex Molecular Systems, Eindhoven University of Technology, Eindhoven, the Netherlands

## Abstract

Membrane binding and unbinding dynamics play a crucial role in the biological activity of several nonintegral membrane proteins, which have to be recruited to the membrane to perform their functions. By localizing to the membrane, these proteins are able to induce downstream signal amplification in their respective signaling pathways. Here, we present a 3D computational approach using reaction-diffusion equations to investigate the relation between membrane localization of focal adhesion kinase (FAK), Ras homolog family member A (RhoA), and signal amplification of the YAP/TAZ signaling pathway. Our results show that the theoretical scenarios in which FAK is membrane bound yield robust and amplified YAP/TAZ nuclear translocation signals. Moreover, we predict that the amount of YAP/TAZ nuclear translocation increases with cell spreading, confirming the experimental findings in the literature. In summary, our in silico predictions show that when the cell membrane interaction area with the underlying substrate increases, for example, through cell spreading, this leads to more encounters between membrane-bound signaling partners and downstream signal amplification. Because membrane activation is a motif common to many signaling pathways, this study has important implications for understanding the design principles of signaling networks.

## Significance

Although it has been shown that membrane localization of signaling proteins can lead to signal amplification, this has not been studied for focal adhesion kinase (FAK) and Ras homolog family member A (RhoA), which are key players in YAP/TAZ signaling. By developing and applying a computational model to various membrane binding scenarios in a realistic cell, we have been able to show that cases with membrane-bound FAK and RhoA yield an amplified YAP/TAZ response downstream of FAK. The results of this study represent an important step toward understanding how FAK membrane binding (dynamics) can affect YAP/TAZ signaling.

## Introduction

Several studies have pointed toward the influence of cell shape and spreading on cell signaling (1, 2, 3, 4, 5, 6, 7). Halder et al. (3) have shown, for example, that irrespective of substrate stiffness, Yes-associated protein (YAP) and its ortholog transcriptional coactivator with PDZ binding motif (TAZ), commonly referred to as YAP/TAZ, were mainly nuclear in spread cells on large adhesive islands. On the other hand, YAP/TAZ was mainly cytoplasmic in round cells on confined adhesive islands. By controlling organ size via the integration of mechanical stimuli (8, 9, 10, 11, 12, 13, 14), YAP and TAZ have been shown to have ubiquitous physiological roles in developmental processes, tissue homeostasis, and malignancy (8, 9, 10, 11, 12, 13, 14).

Importantly, the interactions between cells and their environment occur via transmembrane integrin molecules, of which some induce downstream YAP/TAZ translocation. Recent studies have shown that the integrin family of matrix adhesions is very heterogeneous in terms of size, subcellular distribution, and dynamic composition (15,16). Furthermore, their biological activity is dependent on their interaction with cytoplasmic proteins, which form, via binding and unbinding, the focal adhesion complex (17,18). Binding of the extracellular domains of integrins to their ECM ligands triggers conformational remodeling of their *α* and *β* subunits, which in turn leads to the presentation of binding sites to cytoplasmic proteins (16). Several theoretical and experimental studies have indicated that protein localization to a membrane, by binding to and forming membrane clusters and rafts, helps concentrate the signal to specific areas of the cell membrane and amplify signals from the membrane (19, 20, 21, 22, 23, 24). In particular, the adsorption of a protein to a membrane surface will increase its relative probability of encountering its reaction partner, resulting in an increased reaction rate because of the higher (local) concentration (19). For example, Kholodenko et al. (19) theoretically showed that membrane localization increases the lifetime of complexes formed between signal transduction partners at the membrane, thus leading to an increased downstream activation. In support of this idea, epidermal growth factor receptor (EGFR)-bound Sos (homolog of *Drosophila melanogaster*’s “Son of sevenless” protein) and Ras GTPase-activating protein (RasGAP) lead to a 10^2^- to 10^3^-fold increase in affinity for Ras when restricted to a small volume close to the membrane. This experimental observation has been confirmed by computational modeling (19). Indeed, in the absence of membrane recruitment, to account for the observed activation and deactivation rates of Ras, a 10^2^–10^3^ increase in the Sos and RasGAP cytosolic concentrations would be required (25). Alternatively, the membrane can constrain protein mobility and orientation, again influencing the (local) reaction rate (19). Therefore, understanding how proteins bind and unbind to the membrane and the subsequent induced responses downstream is of great importance to the study of signal transduction. In this study, we want to investigate whether similar mechanisms are at play for the YAP/TAZ signaling pathway.

Given the complexity and number of interactions in the YAP/TAZ signaling pathway, computational modeling is an interesting tool to conduct in silico experiments in a systematic way. More importantly, it allows us to theoretically explore scenarios that are experimentally impossible and as such improve our understanding of the design principles of the signaling networks (26). For example, Spill et al. (27) used an in silico approach to investigate YAP/TAZ stiffness sensing. They showed, among others, that changes in the total focal adhesion kinase (FAK) concentration, which represents the model input signal, resulted in different patterns of YAP/TAZ stiffness response (19). Scott et al. (28) have investigated the effect of substrate dimensionality, i.e., two-dimensional (2D) or three-dimensional (3D) cell-substrate interactions, on YAP/TAZ signaling. Their model results indicated that substrate dimensionality is interpreted differently by the membrane, cytoskeletal, and nuclear modules of the YAP/TAZ signaling cascade because of differences between the surface activation area (i.e., the 2D substrate contact area where FAK is activated) and the membrane reaction area (i.e., the entire plasma membrane area where RhoA binding and downstream reaction occurs). As such, these results highlight that the cell’s surface area available for membrane reactions is an important factor in YAP/TAZ mechanotransduction. Moreover, by altering the diffusivity of FAK and thus the localization of FAK activation, they showed that the nuclear YAP/TAZ fraction increased with increased FAK diffusion coefficient and that this effect is attenuated with increased cell spreading.

In this theoretical study, we aim to build on the above modeling works and investigate the influence of FAK and RhoA localization on YAP/TAZ nuclear translocation. In particular, we implement five different FAK and RhoA activation combination modalities, i.e., cytosolic versus membrane bound, and explore the effect of spatial localization, diffusivity, and membrane (un)binding rates on downstream YAP/TAZ signaling. We seek to understand whether membrane binding has a signaling advantage and help understand the experimental observations reported in literature. We use the YAP/TAZ signaling pathway, for which we extended the well-mixed computational YAP/TAZ model of Sun et al. (27) from a one-dimensional (1D) to a 3D description, similar to Scott et al. (28). Because many signaling pathways are activated at the membrane, understanding their activation pattern and the influence on the downstream signaling thereof is of the highest importance to develop improved cell culture and organoid models as well as advanced regenerative medicine therapies.

## Materials and methods

### YAP/TAZ nuclear translocation model

We studied the relationship between cell shape, cell spreading, and YAP/TAZ nuclear translocation under five different FAK and RhoA activation modalities by using the 1D model of Sun et al. (27), which we extended to three dimensions to include spatial effects by approximating the signaling cascade as a reaction-diffusion system, similar to Scott et al. (28). We performed the implementation in Virtual Cell, a computational and simulation platform (29,30).

**Figure 1:**
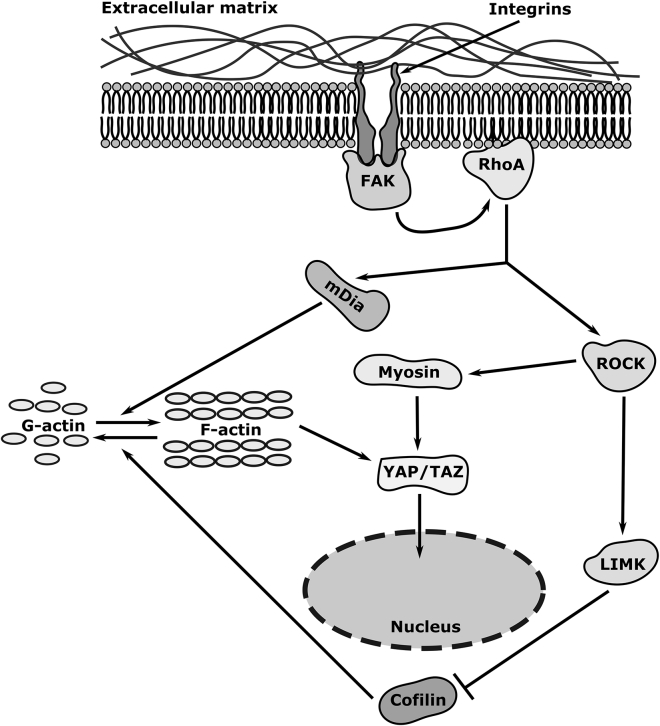
YAP/TAZ nuclear translocation signaling cascade. In our proposed model, the YAP/TAZ signaling cascade is triggered by the activation of FAK at the membrane or in the cytosol (see Fig. 2 for the different activation modalities). The net effect is the phosphorylation of YAP/TAZ under the influence of actomyosin activity, which is in turn translated into YAP/TAZ nuclear translocation. Note that we do not model the entire focal adhesion complex at the membrane but approximate this by FAK activation at the membrane.

**Figure 2:**
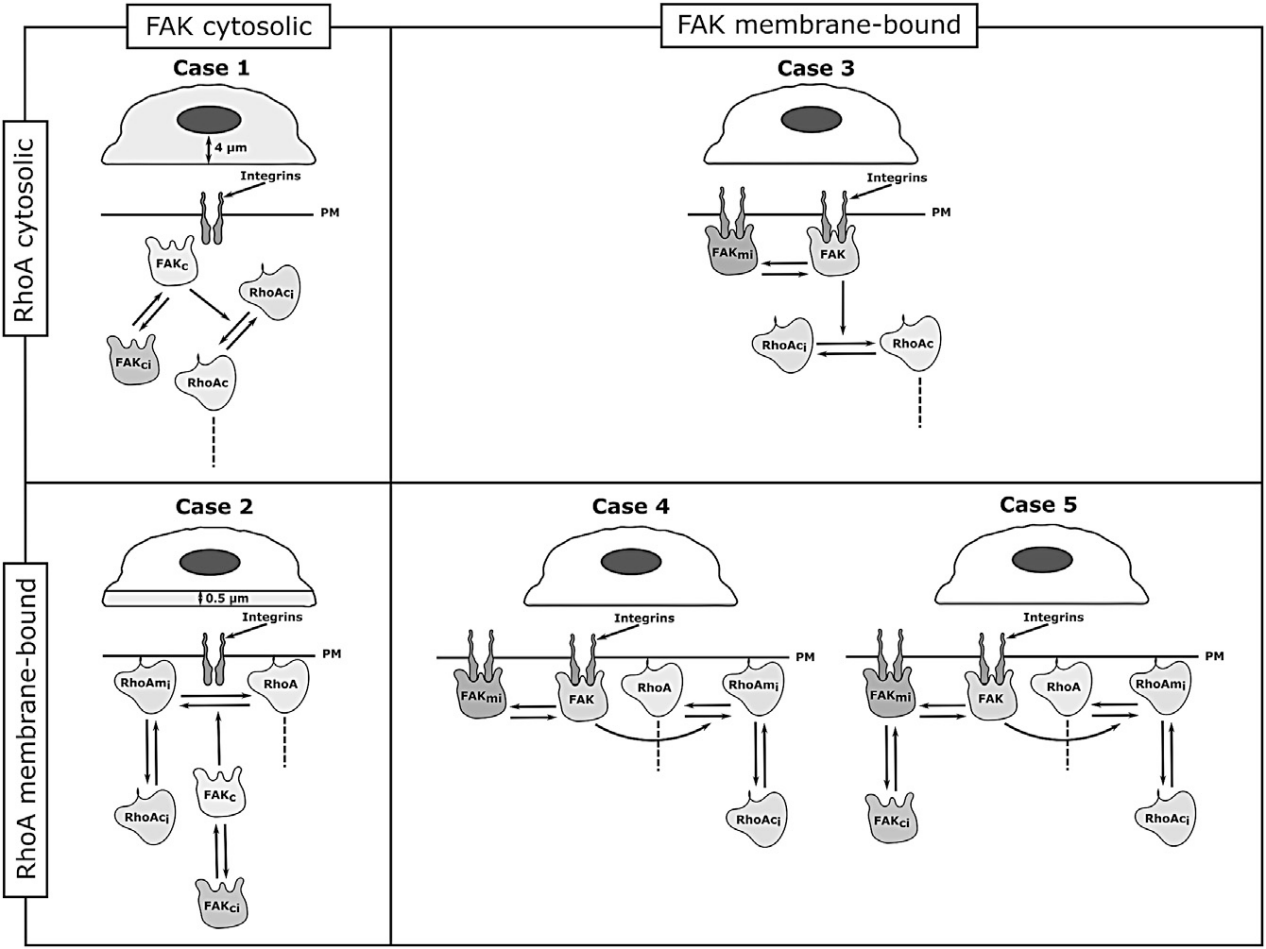
FAK and RhoA activation modalities. Schematic representation of the different cases depicting the FAK and RhoA activation modalities is given. The top part of a quadrant shows the realistic cell shape used in the model; the bottom part shows the signaling pathway, with the dashed line representing the downstream reactions in the YAP/TAZ signaling pathway, here omitted. The subscripts ci and mi denote the inactive cytosolic and membrane-bound forms, respectively, and the active cytosolic, membrane-bound, and cytosolic forms are denoted by c and m, respectively. The inactive forms cycle between inactive cytosolic forms (*FAK*_*ci*_ and *RhoA*_*ci*_ in cases 4 and 5) and inactive membrane-bound forms (*FAK*_*mi*_, exclusively in case 5, and *RhoA*_*mi*_ in cases 4 and 5). Furthermore, downstream signaling always starts from the active RhoA, which can be membrane bound (cases 2, 4, and 5) or cytosolic (cases 1 and 3). The region of active FAK initialization is shown in light grey in the cell outline. PM, plasma membrane.

Briefly, in this work, we model the YAP/TAZ signaling cascade as follows (see Fig. 1). As the initial activation of the signaling cascade, we model the FAK activity and investigate the influence of cytosolic or membrane activation (see Fig. 2) (31). The first downstream effector of FAK is the small GTPase RhoA, whose active form binds to the cell membrane. Activated RhoA can in turn activate mDia and ROCK (32,33). mDia plays a role in stress fiber and filopodia formation and accelerates the elongation of actin filaments (34). ROCK acts on two downstream effectors: myosin and LIM-kinase (LIMK) (35). ROCK favors myosin activity through phosphorylation of its light chain and inhibition of myosin phosphatase (36). The activation of LIMK leads to the inactivation of cofilin, an F-actin cleaving protein (35). This actomyosin activity (i.e., contractility) and stress fiber assembly, as favored by ROCK, translates into YAP/TAZ nuclear translocation (3,8, 9, 10,37).

### In silico model experiments

It has been established that the activation of RhoA occurs at the membrane with the involvement of GEFs (guanine nucleotide exchange factors), guanine nucleotide dissociation inhibitors, and GAPs (GTPase-activating proteins), whereas the inactive RhoA remains cytoplasmic (38, 39, 40, 41). However, recent findings have shown that active and inactive forms of Rho GTPases can coexist on the membrane and can be continuously extracted from the membrane by guanine nucleotide dissociation inhibitors, with the active form being quickly recycled back to the membrane (40), which is captured in the model cases 2, 4, and 5 (see below). Interestingly, overexpression and mutations in the hypervariable regions that are involved in the localization control of the Rho GTPases have been associated with increased cytosolic sequestration (42, 43, 44, 45), which we model in cases 1 and 3 (see below).

Similarly, it has been shown that FAK is activated at the focal adhesion complex and cycles between the membrane and cytoplasm with focal adhesion assembly and disassembly (46,47), which we model in case 5 (see below). On the one hand, point mutations in the FAT domain of FAK can inhibit FAK recruitment to focal adhesions by abrogating FAK-paxillin interaction (48,49), resulting in exclusively cytoplasmic FAK. In addition, other authors have reported that active FAK can be found in the cytoplasm (which we model in cases 1 and 2; see below), and even translocate from the cytoplasm to the nucleus (46,50,51). On the other hand, Emelia and Tumbarello (52) have shown that depletion of FIP200 and loss of Atg5 (two autophagosome regulators) lead to retention of active FAK at focal adhesions, which we model in cases 3 and 4 (see below).

Importantly, the particular localization of FAK or RhoA has been shown to influence downstream YAP/TAZ signaling. For example, Valon et al. (53) showed that plasma membrane localization of an engineered optogenic RhoA activator (optoGEF-RhoA) leads to YAP nuclear localization, whereas mitochondrial localization is associated with a decrease in nuclear YAP. Similarly, lovastatin, a small molecule that prevents membrane localization of RhoA, was found to inhibit the nuclear localization of YAP (54,55). Moreover, alternative splicing of FAK, shown to result in reduced focal adhesion formation, increased the cytoplasmic localization of FAK and reduced nuclear YAP (56,57).

In summary, to adequately compare the effect of cytoplasmic and membrane localization of RhoA and FAK on the YAP/TAZ nuclear translocation response and based on the previous observations, each of the following five model cases was chosen such that we could progressively build from an all-cytoplasmic localization of RhoA and FAK to different scenarios of membrane localization (see Fig. 2).

1. Case 1: FAK and RhoA are cytosolic and get activated in the cytoplasm. They both freely diffuse within the cytoplasm.
2. Case 2: FAK is cytosolic and RhoA is membrane bound (part of the focal adhesion complex); FAK is activated in a small region (0.5 *μ*m in height) close to the membrane at the base of the cell.
3. Case 3: FAK is membrane bound and does not diffuse within the membrane (part of the focal adhesion complex). RhoA is cytosolic and freely diffuses within the cytoplasm.
4. Case 4: FAK and RhoA are both membrane bound (part of the focal adhesion complex), and RhoA can bind and unbind from the focal adhesion complex. Additionally, FAK does not diffuse within the focal adhesion complex, but RhoA can diffuse within the membrane and in the cytoplasm.
5. Case 5: FAK and RhoA are both membrane bound (part of the focal adhesion complex); they can both bind and unbind from the focal adhesion complex. FAK does not diffuse within the focal adhesion complex, whereas RhoA can diffuse within the membrane. Both FAK and RhoA diffuse within the cytoplasm.

The 0.5 *μ*m band in case 2 is used to represent a very small zone of activation of FAK close to the membrane, which models the focal adhesion region without explicitly segregating FAK from the cytosol. We chose 0.5 *μ*m because it was the smallest distance that could be achieved at the mesh size we used while keeping the same initial FAK number of molecules across cases.

### Model equations

In the computational model, all proteins have an active and inactive form; G-actin represents the inactive form of F-actin. The active form represents the phosphorylated state, except for cofilin, which gets activated upon dephosphorylation (35). The dynamics of the active and inactive forms of the signaling molecules are described in general terms in Eqs. 1 and 2.

Active forms (general equation) are as follows:

(1) $$\frac{\partial S}{\partial t}=Q_{s_{i}}\times S_{i}-d_{S}\times S+D_{s}\times \nabla^{2}S.$$

The active protein is denoted with *S*, and the inactive is denoted *S*_*i*_. $Q_{S_{i}}$ represents the activation rate of the inactive form, and *d*_*S*_ represents the degradation rate of the active form (in both Eqs. 1 and 2). *D*_*s*_ represents the diffusion coefficient.

Inactive forms (general equation) are as follows:

(2) $$\frac{\partial S_{i}}{\partial t}=d_{S}\times S-Q_{S_{i}}\times S_{i}+D_{S_{i}}\times \nabla^{2}S_{i}.$$

The equations for the dynamics of specific proteins presented in Eqs. 3, 4, 5, 6, 7, 8, 9, 10, 11, 12, 13, 14, and 15 focus on the active forms and are adapted from Sun et al. (27). Because we focus on YAP/TAZ signaling resulting from FAK activation only, we removed the baseline activation of RhoA, Myosin, LIMK, and F-actin by other pathways, i.e., independent from the FAK signaling cascade, as well as the LATS-related terms (LATS_0_ and LATSp) and the constitutive baseline activation of YAP/TAZ (*K*_*CN*_). All the parameter values are found in Table 1, and the equations are further detailed below.

**Table 1:** Parameter settings

Parameter	Definition	Value	References
*K*_*fρ*_	FAK-dependent RhoA phosphorylation	9 s^−2^	(27)
*mDia*_*b*_	mDia threshold	0.13 *μ*M	(27)
*K*_*dρ*_	RhoA dephosphorylation	0.625 s^−1^	(58)
*ROCK*_*b*_	ROCK threshold	0.26 *μ*M	(27)
*LD*	ligand density	2	(59)
*C*	ligand density × Emol when the FAK activation is *K*_*sf*_/2	45 *μ*M	(27)
*K*_*df*_	FAK dephosphorylation rate	0.035 s^−1^	(59)
*K*_*sf*_	FAK activation rate due to integrin activation	0.3795 s^−1^	(59)
*K*_*rρ*_	RhoA-dependent ROCK activation	2.2 s^−1^*μ*M^−1^	(60,61)
*Kd*_*rock*_	ROCK degradation rate	0.8 s^−1^	(27)
*K*_*lr*_	ROCK-dependent LIMK activation rate	0.07 s^−1^	(27)
*K*_*dl*_	LIMK degradation rate	2 s^−1^	(27)
*τ*	ROCK-dependent LIMK activation amplification	200 *μ*M^−1^	(27)
*K*_*dm*_	mDia degradation rate	1 s^−1^	(27)
*K*_*mρ*_	RhoA-dependent mDia activation	1 s^−1^*μ*M^−1^	(27)
*K*_*my*_	myosin activation rate	0.015 s^−1^	adapted from (27)
*K*_*dmy*_	myosin degradation rate	0.067 s^−1^	(62)
*ε*	ROCK-dependent myosin activation	40 *μ*M^−1^	(27)
*E*	stiffness of substratum	10^6^ kPa	(63,64)
*K*_*turnover*_	cofilin dephosphorylation rate	0.04 s^−1^	(65)
*K*_*cl*_	LIMK-dependent cofilin phosphorylation rate	0.7 *μ*M^−2^ s^−1^	(41)
*α*	mDia-dependent F-actin activation amplification	40 *μ*M^−1^	(27)
*K*_*dep*_	F-actin depolymerization rate	3.5 s^−1^	(66)
*K*_*dfc*_	cofilin-dependent F-acting severing rate	8 s^−1^*μ*M^−1^	(27)
*K*_*f*_	F-actin polymerization rate	0.4 s^−1^	(27)
*K*_*CN*_	YAP/TAZ nuclear import rate	0.4 s^−1^	fitted
*K*_*cy*_	cytoplasmic YAP/TAZ phosphorylation rate	20 *μ*M^−1^ s^−1^	(27)
*K*_*dcy*_	cytoplasmic YAP/TAZ dephosphorylation rate	0.1 *μ*M^−1^ s^−1^	(27)
*KF*_*on*_	FAK membrane binding rate	0.029 s^−1^	(18)
*KF*_*off*_	FAk membrane unbinding rate	0.017 *μ*m^−1^ s^−1^	(18)
*K*_*on*_	RhoA membrane binding rate	20 s^−1^	estimated
*K*_*off*_	RhoA membrane unbinding rate	0.5 *μ*m^−1^ s^−1^	estimated
*D*_*FAKci*_	diffusion coefficient of inactive cytosolic FAk	15.96 *μ*m^2^ s^−1^	calculated
*D*_*RhoAci*_	diffusion coefficient of inactive cytosolic RhoA	28.03 *μ*m^2^ s^−1^	calculated
*D*_*RhoA*_	diffusion coefficient of active RhoA	0.06 *μ*m^2^ s^−1^	calculated
*D*_*ROCK*_	diffusion coefficient of ROCK	11.39 *μ*m^2^ s^−1^	calculated
*D*_*m*_	diffusion coefficient of mDia	15.16 *μ*m^2^ s^−1^	calculated
*D*_*my*_	diffusion coefficient of myosin	9.76 *μ*m^2^ s^−1^	calculated
*D*_*LIMK*_	diffusion coefficient of LIMK	18.82 *μ*m^2^ s^−1^	calculated
*D*_*c*_	diffusion coefficient of cofilin	29.44 *μ*m^2^ s^−1^	calculated
*D*_*Fcyto*_	diffusion coefficient of F-actin	0.001 *μ*m^2^ s^−1^	calculated
*D*_*G-actin*_	diffusion coefficient of G-actin	22.58 *μ*m^2^ s^−1^	calculated
*D*_*YAPTAZc*_	diffusion coefficient of cytoplasmic active YAP/TAZ	20.71 *μ*m^2^ s^−1^	calculated

The model equations Eqs. 3, 4, 5, 6, 7, 8, 9, 10, 11, 12, 13, 14, and 15 describe the standard scenario (case 5). In this scenario, both FAK and RhoA can cycle between their membrane-bound forms and their cytosolic freely diffusing forms. Inspired by Spill et al. (67) and Holmes et al. (68), we distinguish between membrane-bound inactive FAK (*FAK*_*mi*_) and RhoA (*RhoA*_*mi*_), membrane-bound active FAK (*FAK*) and RhoA (*RhoA*), and cytosolic inactive FAK (*FAK*_*ci*_) and RhoA (*RhoA*_*ci*_). Importantly, although we acknowledge the complex and dynamic composition of the focal adhesion complex, we approximate it here by a FAK activation at the membrane.

Inactive cytosolic FAK (*FAK*_*ci*_) diffuses in the cytosol with a diffusion coefficient $D_{FAK_{ci}}$ (Eq. 3). It then binds to the membrane in a reversible manner at respective binding and unbinding rates *KF*_*on*_ and *KF*_*off*_ (see Boundary conditions). The membrane-bound form of inactive FAK (*FAK*_*mi*_) results from the inactivation of the active bound form of FAK (*FAK*). The YAP/TAZ signaling cascade is initiated by setting a predetermined initial amount of active FAK, which then decays with time (Eq. 5).

*FAK*_*ci*_:

(3) $$\frac{\partial FAK_{ci}}{\partial t}=D_{FAK_{ci}}\times \nabla^{2}FAK_{ci}.$$

*FAK*_*mi*_:

(4) $$\frac{\partial FAK_{mi}}{\partial t}=K_{df}\times FAK+D_{FAK_{mi}}\times \nabla^{2}FAK_{mi}.$$

*FAK:*

(5) $$\frac{\partial FAK}{\partial t}=-K_{df}\times FAK+D_{FAK}\times \nabla^{2}FAK.$$

Just like the inactive cytosolic FAK, the inactive cytosolic RhoA (*RhoA*_*ci*_) only diffuses in the cytosol with diffusion coefficient $D_{RhoA_{ci}}$ (Eq. 6).

*RhoA*_*ci*_ *:*

(6) $$\frac{\partial RhoA_{ci}}{\partial t}=D_{RhoA_{ci}}\times \nabla^{2}RhoA_{ci}.$$

*RhoA*_*mi*_:

(7) $$\frac{\partial RhoA_{mi}}{\partial t}=K_{d\rho }\times RhoA-K_{f\rho }FAK^{2}\times RhoA_{mi}+D_{RhoA_{mi}}\times \nabla^{2}RhoA_{mi}.$$

Inactive cytosolic RhoA binds to the membrane with a rate *K*_*on*_ (see Boundary conditions). The inactive membrane-bound RhoA unbinds at a rate *K*_*off*_, diffuses on the plasma membrane with a rate *D*_*RhoAmi*_, gets activated by FAK at a rate *K*_*fρ*_, and degrades at a rate *dK*_*dρ*_ (Eq. 8).

*RhoA*:

(8) $$\frac{\partial RhoA}{\partial t}=K_{f\rho }FAK^{2}RhoA_{mi}-K_{d\rho }RhoA+D_{RhoA}\nabla^{2}RhoA.$$

Note that the inactive form of RhoA is assumed to diffuse 500 times faster in the cytoplasm than the active membrane-bound form (see Table 1) to capture the relative immobility of the proteins in the focal adhesion complex.

Downstream of RhoA, there are several cytoskeletal regulators whose dynamics are described from Eqs. 9, 10, 11, 12, 13, 14, and 15. RhoA activates mDia and ROCK (32,33). ROCK is a cystoskeleton-associated protein kinase involved in cell shape regulation and mDia is involved in accelerating the actin polymerization rate (5–15 times) (27,34).

*ROCK*:

(9) $$\frac{\partial ROCK}{\partial t}=-K_{drock}\times ROCK+D_{ROCK}\times \nabla^{2}ROCK.$$

In Eq. 9, *K*_*drock*_ is the degradation rate of ROCK, and *D*_*ROCK*_ is the diffusion coefficient of ROCK.

*mDia*:

(10) $$\frac{\partial mDia}{\partial t}=-K_{dm}\times mDia+D_{m}\times \nabla^{2}mDia.$$

In Eq. 10, *K*_*dm*_ is the degradation rate of mDia and *D*_*m*_ the diffusion coefficient of mDia. It should be noted that the inactive forms of ROCK and mDia get activated by interacting with active RhoA at the membrane (see Boundary conditions). Active ROCK and mDia then diffuse from their activation at the plasma membrane into the cytoplasm. Also, other activation modes have been explored; see the section In silico model experiments as well as Fig. 2.

ROCK acts on two downstream effectors; myosin and LIMK. ROCK favors myosin activity through phosphorylation of its light chain and inhibition of myosin phosphatase (35).

*Myo*:

(11) $$\frac{\partial Myo}{\partial t}=K_{my}\times \varepsilon \times T_{ROCK}\times Myo_{i}-K_{dmy}\times Myo+D_{my}\times \nabla^{2}Myo.$$

The activation of myosin is represented by *K*_*my*_, the degradation rate is *K*_*dmy*_, and its diffusion coefficient is *D*_*my*_ (Eq. 11). Here, *ε* is the active myosin amplification rate by active ROCK.

*LIMK*:

(12) $$\frac{\partial LIMK}{\partial t}=K_{lr}\times \tau \times T_{ROCK}\times LIMK_{i}-K_{dl}\times LIMK+D_{LIMK}\times \nabla^{2}LIMK.$$

The activation rate of LIMK is *K*_*lr*_, and *K*_*dl*_ is the degradation rate and *D*_*LIMK*_ the diffusion coefficient (Eq. 12). *T*_*ROCK*_ is the corresponding threshold function for ROCK (see below). The activation of LIMK leads to the inactivation of cofilin, an F-actin cleaving protein.

*Cofilin*:

(13) $$\frac{\partial Cofilin}{\partial t}=K_{turnover}\times Cofilin_{i}-K_{cl}\times LIMK^{2}\times Cofilin+D_{c}\times \nabla^{2}Cofilin.$$

In Eq. 13, *K*_*turnover*_ is the cofilin activation rate, *K*_*cl*_ is the cofilin deactivation rate, and *D*_*c*_ is the diffusion coefficient of cofilin. mDia and cofilin are involved in the assembly and disassembly of filamentous actin from and to globular G-actin subunits, respectively.

Cytosolic F-actin:

(14) $$\frac{\partial Fcyto}{\partial t}=K_{f}\times \alpha \times T_{mDia}\times mDia\times Gactin-K_{dep}\times Fcyto-K_{dfc}\times Cofilin\times Fcyto+D_{Fcyto}\times \nabla^{2}Fcyto.$$

In Eq. 14, *K*_*f*_ is the assembly rate, *K*_*dep*_ the depolymerization rate, *K*_*dfc*_ the disassembly rate, and *D*_*Fcyto*_ the diffusion coefficient of F-actin. *T*_*mDia*_ is the corresponding threshold function for mDia (see below). G-actin, considered as the inactive form of F-actin, diffuses with diffusion coefficient 22.58 *μ*m^2^ s^−1^, estimated from the Stokes-Einstein relation (69). Here, we assign an arbitrary low diffusion coefficient (i.e., 0.001 *μ*m^2^ s^−1^) to F-actin because it is a filamentous protein made up of G-actin subunits.

Nuclear translocation of YAP/TAZ depends on actomyosin activity (i.e., contractility) and stress fiber assembly, as favored by ROCK (27). To capture these mechanochemical effects, we implemented Eq. 15, in which *Fcyto* and *Myo* influence the activation of the inactive cytosolic YAP/TAZ. The active cytosolic YAP/TAZ subsequently translocates into the nucleus. As such, we distinguish between inactive cytoplasmic YAP/TAZ (*YAPTAZ*_*ci*_), active cytoplasmic YAP/TAZ (*YAPTAZ*_*c*_), and nuclear YAP/TAZ (*YAPTAZ*_*n*_) which refers to the amount of *YAPTAZ*_*c*_ which is shuttled in the nucleus (see Boundary conditions; Eqs. 15 and 24).

*YAPTAZ*_*c*_ (active cytoplasmic YAP/TAZ):

(15) $$\frac{\partial YAPTAZ_{c}}{\partial t}=K_{cy}\times Fcyto\times Myo\times YAPTAZ_{ci}-K_{dcy}\times YAPTAZ_{C}+D_{YAPTAZ_{C}}\times \nabla^{2}YAPTAZ_{c}.$$

In Eq. 15, inactive YAP/TAZ (*YAPTAZ*_*ci*_) gets activated by phosphorylation at a rate *K*_*cy*_ and gets deactivated by dephosphorylation at a rate *K*_*dcy*_. $D_{YAPTAZ_{C}}$ is the diffusion coefficient of active cytosolic YAP/TAZ. The notion that ROCK and mDia concentrations have to exceed a threshold value to trigger LIMK and G-actin activation, respectively, is approximated by a threshold T function (Eqs. 16 and 17), similar to (27). The linear region of the T function corresponds to a scenario in which the ROCK or mDia concentration value is above *ROCK*_*B*_ or *mDia*_*B*_.

(16) $$T_{ROCK}\;=\left\{ \begin{array}{l} 0\;when\;ROCK\leq ROCK_{B}\\ ROCK-ROCK_{B}\;when\;ROCK>ROCK_{B} \end{array}\right..$$

(17) $$T_{mDia}=\left\{ \begin{array}{l} 0\;when\;mDia\leq mDia_{B}\\ mDia-mDia_{B}\;when\;mDia>mDia_{B} \end{array}\right..$$

In the results, we calculate the YAP/TAZ nuclear fraction (YTNF) as follows:

$$\text{YTNF}=\frac{YAPTAZ_{n}\left(number\;of\;molecules\right)}{YAPTAZ_{c}+YAPTAZ_{ci}+YAPTAZ_{n}\left(number\;of\;molecules\right)\;}.$$

### Boundary conditions

The boundary conditions represent mathematically what happens at the boundaries of the specified domain, i.e., whether components can enter or leave the system or move from one domain (e.g., the cytoplasm) to another (e.g., the nucleus).

#### Boundary condition at the plasma membrane for FAK, RhoA, ROCK, and mDia

The boundary condition for *FAK*, for case 5 (see Eq. 18 below), at the plasma membrane is such that the (un)binding events are in balance with the diffusive flux:

(18) $$-D_{FAK_{ci}}\times e_{n}\times \nabla_{V}FAK_{ci}=\left(KF_{on}\times FAK_{ci}-N\times KF_{off}\times FAK_{mi}\right).$$

For *RhoA* in cases 2, 4, and 5, we have a boundary condition as follows:

(19) $$-D_{RhoA_{ci}}\times e_{n}\times \nabla_{V}RhoA_{ci}=\left(K_{on}\times RhoA_{ci}-N\times K_{off}\times RhoA_{mi}\right),$$

where *V* is the cytosol domain, *e*_*n*_ the unit outward normal vector at the membrane, and the terms *e*_*n*_ × $\nabla_{V}$*FAK*_*ci*_ and *e*_*n*_ × $\nabla_{V}$ *RhoA*_*ci*_ the projection of the gradient of *FAK*_*ci*_ and *RhoA*_*ci*_ on the unit normal vector on the surface, similar to Spill et al. (67).

In case 3, *RhoA*_*ci*_ has the following boundary condition because it gets activated by interacting with active FAK at the membrane:

(20) $$-D_{RhoA_{ci}}\times e_{n}\times \nabla_{V}RhoA_{ci}=N\times K_{f\rho }\times FAK^{2}\times RhoA_{ci}.$$

*ROCK* and *mDia* have the following boundary conditions in cases 2, 4, and 5 because they get activated by interacting with active RhoA at the membrane:

(21) $$-D_{ROCK_{i}}\times e_{n}\times \nabla_{V}ROCK_{i}=\;N\times \left(K_{r\rho }\times RhoA\times ROCK_{i}\right)$$

and

(22) $$-D_{mDia_{i}}\times e_{n}\times \nabla_{V}mDia_{i}=N\times \left(K_{m\rho }\times RhoA\times mDia_{i}\right),$$

where *K*_*rρ*_ is the activation rate of ROCK by RhoA and *K*_*mρ*_ refers to the activation rate of mDia by RhoA. It is worth noting that a conversion factor, *N*, was used for the boundary conditions described in Eqs. 18, 19, 20, 21, and 22. This *N* term was used to convert from volume units to membrane units, and it embodies the length scale difference between membrane and cell compartments (*N* = volume of cell/surface area of cell) as described in (28,67). In the Virtual Cell environment, this conversion is handled internally.

#### At the plasma membrane-cytoplasm boundary for all other variables

For all the components outside of the cases mentioned above, a no-flux boundary condition is valid at the plasma membrane.

#### Cytoplasm-nucleus boundary for YAP/TAZ_c_

For all the components, a no-flux boundary condition is valid at the cytoplasm-nucleus boundary, except for YAP/TAZ, which can move into the nucleus (see Eq. 23). Note that the computational analysis focuses on the YAP/TAZ input, and thus, the model does not include an export term.

#### Boundary condition at the nuclear membrane

(23) $${\;\frac{\partial YAPTAZ_{c\;}}{\partial t}|}_{NM}=-K_{CN}YAPTAZ_{c},$$

with NM the nuclear membrane.

### Initial conditions and diffusion coefficients

The diffusion coefficients are as shown in Table 1. Note that the active and inactive forms have the same diffusion coefficients except otherwise mentioned. We assume that FAK diffuses in the cytosol, but not on the plasma membrane. The values for the diffusion coefficients were obtained from the Stokes-Einstein equation (69) (details in Supporting materials and methods), and we assigned the same calculated value of the diffusion coefficient to the inactive and active forms for all species that we considered unbound to the plasma membrane when activated. For RhoA, which we considered bound to the membrane in cases 3–5, and based on Marée et al. (2), we modeled diffusion such that the cytosolic inactive RhoA diffused 500 times faster than its active membrane-bound counterpart (active RhoA). This difference between membrane-bound and cytosolic diffusivities is based on the observations of Ueda et al. (70) on G-protein kinetic in chemotactic signaling and the estimations of Postma et al. (71), who showed that membrane-bound proteins were able to diffuse much faster in the cytosol than when bound to the plasma membrane. In addition, by using expectation maximization on in vivo membrane-bound Rho GTPase data from single-molecule tracking photoactivated microscopy, Koo et al. (72) identified various Rho GTPases diffusion coefficients ranging from 0.0007 *μ*m^2^ s^−1^ to around 0.7 *μ*m^2^ s^−1^, which supports the value used in this study (0.06 *μ*m^2^ s^−1^). The discrepancy between membrane-bound and cytosolic diffusivities could be explained by several factors inherent to the plasma membrane dynamics, including diffusion attenuation structures such as the cytoskeleton meshwork, the existence of lipid microdomains and rafts, and protein-protein interactions (73, 74, 75). Note should be taken that because F-actin is a filamentous protein, it was assigned a very low diffusion coefficient of 0.001 *μ*m^2^ s^−1^. The diffusion coefficient of YAP/TAZ was determined from the average of the molecular weight of YAP and TAZ.

The initial concentrations are as shown in Table 2, with all species in the YAP/TAZ signaling pathway initially inactive, except for FAK. The FAK input is such that the initial number of active and inactive FAK molecules are kept constant across all experiments. This initial amount of active FAK is set to match the amount of active FAK in (28), and we fitted the YAP/TAZ nuclear import rate to obtain a YAP/TAZ nuclear fraction (YTNF) of 80% for case 5 of the FAK activation mode (see In silico model experiments), similar to what is reported in literature (76) for a stiffness value classified as “high” (23 kPa) for a similar cell volume as the standard cell in this study (base radius 16 *μ*m). To find the best fitting import rate, we varied this parameter while keeping all other parameter values at their standard values as described in Tables 1 and 2.

**Table 2:** Initial concentrations used in the YAP/TAZ nuclear translocation model

Species	Inactive	Active
*FAK*_*ci*_^a^	1,260,018.86 molecules (0.75–2.0 *μ*M)	–
*FAK*_*mi*_	0 *μ*M	–
*FAK*^b^	–	58,104.35 molecules (0.035–0.25 *μ*M)
*Cofilin*	1 *μ*M	0 *μ*M
*RhoA*_*ci*_	1 *μ*M	–
*RhoA*_*mi*_	0 *μ*M	–
*RhoA*	–	0 *μ*M
*LIMK*	1 *μ*M	0 *μ*M
*Actin*	0 *μ*M (G-actin)	1 *μ*M (F-actin)
*YAP/TAZci*	1 *μ*M	–
*YAP/TAZc*	–	0 *μ*M
*YAP/TAZn*	–	0 *μ*M
*FAK*	3.5 *μ*M	0 *μ*M
*mdia*	1 *μ*M	0 *μ*M
*ROCK*	1 *μ*M	0 *μ*M
*myosin*	1 *μ*M	0 *μ*M

Note that G-actin is the inactive form of F-actin. Note that when Virtual Cell determines the number of molecules, based on the domain volume and specified concentration, it does not round off to the nearest whole number.

a The initial number of molecules of inactive (cytosolic) form correspond to 0.75 *μ*M in cases 1, 2, and 5 and 2.0 *μ*M in cases 3 and 4 for a standard cell of base radius 16 *μ*m.

b The number of molecules of the active form correspond to a concentration of 0.035 *μ*M for case 1, 0.36 *μ*M for case 2, and 0.25 *μ*M for cases 3–5. The values are within similar ranges for total FAK or signaling protein initial FAK concentration (27,28,77, 78, 79).

In this study, we contrast two types of initial FAK activation. In a first approach, we trigger the YAP/TAZ signaling dynamics by defining a predetermined initial amount of active (and inactive) FAK (see Table 2). In the second approach, inactive FAK is activated at a particular activation rate for 100 s, including the effect of substrate stiffness (see Eq. S2). As such, initially, there is no active FAK, and the total amount of inactive FAK is set equal to the total amount of FAK (active plus inactive) of the first modality.

### Geometry

We obtained a previously defined theoretical realistic cell shape (80) in Virtual Cell, in which we implemented our in silico experiments. This geometry was also used by Scott et al. (28), which will enable easier comparison. Although this is an approximate geometry, its analytical expression is shown to best approximate a fibroblast with a discoid base as presented in Schneider and Haugh (81). Experiments were performed for all five cases for spreading cells with four base cell radii of 14, 16, 18, and 20 *μ*m. The standard cell has a base radius of 16 *μ*m.

In all instances, the cell volume was kept constant at 2925 *μ*m^3^, the distance from the nucleus to the cell membrane at the base was 4 *μ*m, and the nuclear volume was 125 *μ*m^3^. The nucleus was centered with respect to the base of the cell (Fig. S1).

### Numerical implementation

In Virtual Cell, we used the fully implicit finite volume regular grid solver with a variable time step (range 0–0.1 s) to find numerical solutions to the partial differential equations describing the signaling cascade. This solver uses the finite volume method to represent partial differential equations as algebraic discretization equations, which exactly preserves conservation laws and employs a Sundials stiff solver CVODE for time stepping (method of lines). The values are calculated at discrete places on a meshed geometry. We used a 3D grid with 80 × 33 × 25 elements (1 element = 0.58 × 0.69 × 0.67 *μ*m) with absolute tolerance 10^−9^ and relative tolerance of 10^−7^. A Virtual Cell mesh is a set of discrete elements (here 3D) defining the spatial domain on which the mathematical operations of spatial solvers occur. Virtual Cell meshes are regular grids created by dividing space (geometry size) in each dimension for forming a lattice of cells (mesh size). We performed a mesh convergence analysis and selected this mesh size as a good tradeoff between simulation time and accuracy (results not shown). We used the high-performance computing infrastructure of the Center for Cell Analysis and Modeling of the University of Connecticut Health campus at Farmington, CT, to remotely run our simulations in Virtual Cell. All the simulation code is available in the Virtual Cell repository; see Supporting materials and methods for details.

## Results

### Membrane localization of FAK is important for YAP/TAZ nuclear translocation

Using the computational model developed above, we explored how different FAK and RhoA activation scenarios, as well as cell shape and spreading, influence YAP/TAZ nuclear translocation. Experimental and computational evidence has demonstrated that YAP/TAZ nuclear translocation is influenced by cell shape (1, 2, 3, 4, 5, 6, 7,27); here, we asked how nuclear translocation is transiently influenced by the activation mode, i.e., cytosolic (cases 1 and 2) versus membrane-bound activation (cases 3–5).

**Figure 3:**
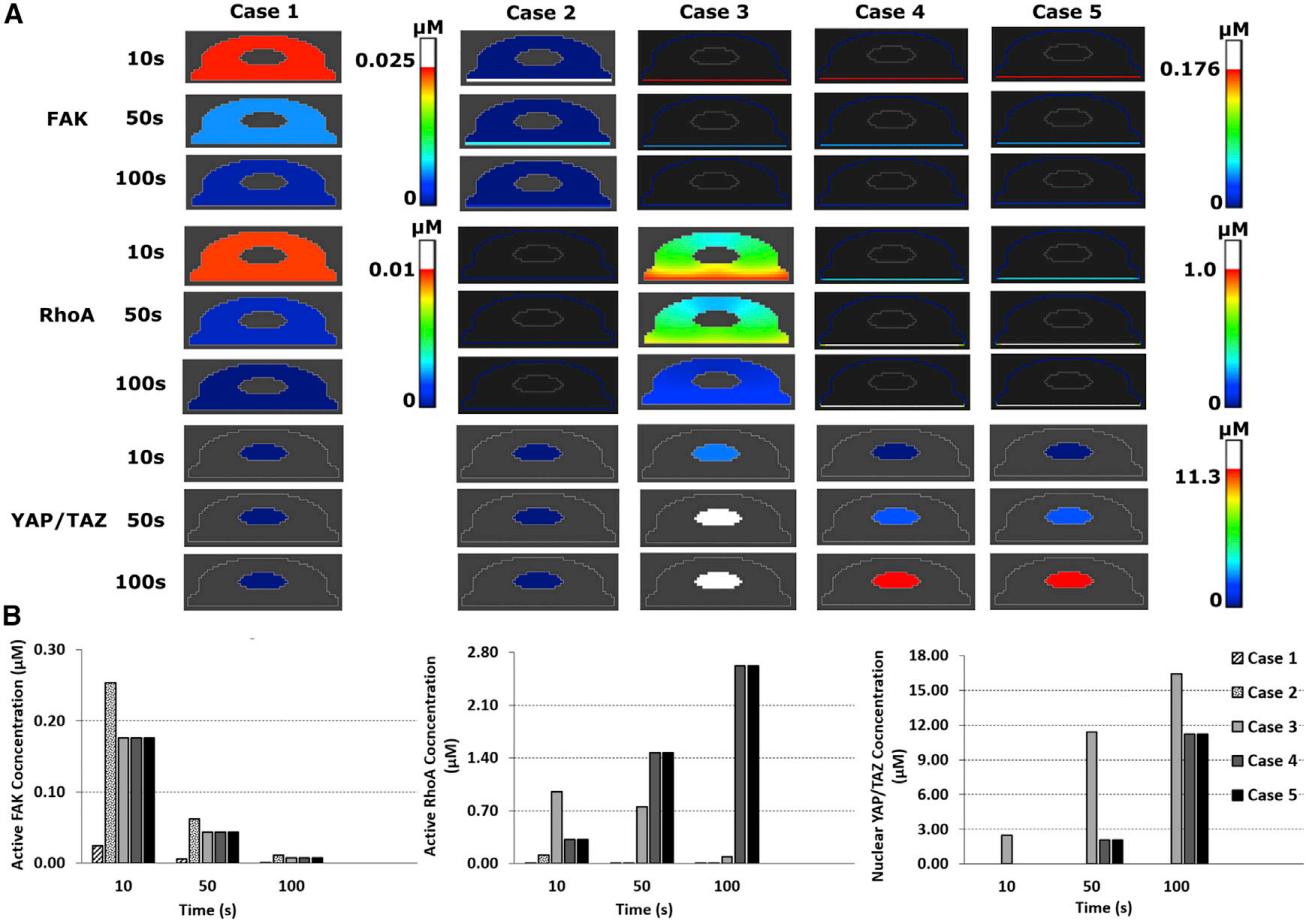
Overview of spatiotemporal predictions of the YAP/TAZ model for cases 1–5. Realistic cells with base radii 16 *μ*m for the five cases depict the evolution of active FAK, RhoA, and nuclear YAP/TAZ for three time points. (*A*) Gradient profiles within the cells (*B*) temporal evolution of concentrations of active FAK, RhoA and nuclear YAP/TAZ. The sampled point is located in the cell membrane in the middle of the cell base for FAK in cases 3, 4, and 5 and for RhoA in cases 2, 4, and 5. For FAK in cases 1 and 2 and RhoA in cases 1 and 3, the point is cytoplasmic and in the middle of the base of the cell base. The coordinate values of the sampling points are supplied in the Table S1. In case 2, the white line represents the local FAK concentration, as in this case FAK is activated in a small region (0.5 *μ*m in height) close to the membrane at the base of the cell (see also Fig. 2). Note that for FAK and RhoA, two scale bars are included to improve the presentation of the results, i.e., for case 1 the scale bars are to the right of the results of case 1, whereas for cases 2–5 the results are to the right of the results of case 5. The white regions correspond to values above the highest value (*red*) indicated on the scale bars. To see this figure in color, go online.

Fig. 3 shows an overview of the spatiotemporal dynamics of FAK, RhoA, and YAP/TAZ for the different activation modes, in which the total initial amount of FAK is kept constant for comparison. Downstream of FAK, RhoA gets activated, which in turn activates downstream signaling components, resulting ultimately in YAP/TAZ nuclear translocation (see also Fig. 1). Interestingly, for cases 1 and 2, in which FAK is initially activated in the cytosol (case 1) or in a region close to the membrane (case 2), there is no YAP/TAZ nuclear translocation (0 *μ*M) in comparison to cases 3–5, in which FAK is activated on the plasma membrane, resulting in 16.44 *μ*M nuclear YAP/TAZ concentration for case 3 and 11.23 *μ*M for cases 4 and 5 at 100 s. Because of differences in dimensionality of the membrane (2D) and cytoplasm (3D), the same initial amount of FAK molecules translates into a higher initial FAK concentration at the membrane and thus higher downstream activation rates and ultimately YAP/TAZ nuclear concentrations for cases 3–5. Indeed, for the standard cell size and initial number of FAK molecules, when RhoA and FAK do not colocalize at the membrane through (un)binding, it is impossible to trigger strong enough signals downstream to exceed the ROCK and mDia thresholds (see Eqs. 16 and 17). However, higher amounts of initial active FAK do result in YAP/TAZ nuclear translocation for cases 1 and 2 (see Fig. S6). Another unexpected observation relates to the fact that in cases 4 and 5, the active RhoA concentration continues to increase over time, despite active FAK decreasing over time, whereas in case 3 the active RhoA returns to zero together with FAK (even though the activation and deactivation rate of RhoA is the same for all cases). Indeed, because of the particular membrane binding and unbinding dynamics of RhoA in cases 4 and 5, including cytosolic inactive, membrane-bound inactive, and membrane-bound active species, the amount of inactive RhoA available for activation at the membrane changes. Moreover, in cases 4 and 5, both RhoA and FAK are membrane bound and thus in close proximity. As such, the active RhoA gradually rises in cases 4 and 5, whereas the inactive RhoA is almost immediately activated in case 3 (see also Fig. 5 *B* below). In summary, these results are in line with the known relevance of membrane protein localization for downstream signal amplification.

### YAP/TAZ nuclear translocation increases with cell spreading when RhoA is membrane bound

**Figure 4:**
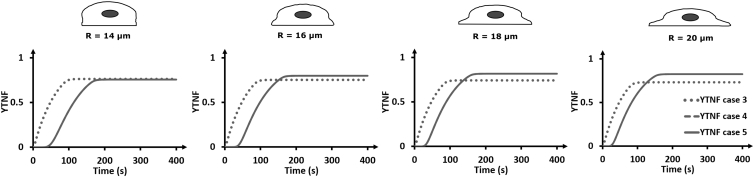
Influence of cell spreading and activation mode on YAP/TAZ nuclear translocation. The dotted line represents the YTNF as a function of time for case 3. The full line represents the YTNF as a function of time for cases 4 and 5 because they overlap. Fig. S2 compares the steady-state YTNF with cell spreading across the three cases in more detail.

Because YAP/TAZ has been shown to be principally nuclear in spread cells compared to confined cells where YAP/TAZ is located in the cytoplasm (3,8), we next explored the effect of cell spreading under the various FAK activation modes as described earlier (cases 3–5). We focus here on cases 3–5, as cases 1 and 2 did not show YAP/TAZ nuclear translocation at standard settings. Fig. 4 shows that the YAP/TAZ fraction increases with time for all activation modes, although for case 3 the nuclear fraction starts to increase earlier and reaches the steady-state concentration faster than for cases 4 and 5. For cases 4 and 5, there was also an increase in steady-state YAP/TAZ nucclear fraction (YTNF) with increased cell spreading (Fig. 4). For case 3, in which FAK is membrane bound and RhoA is cytosolic, the YTNF reduced slightly with cell spreading (from 0.76 for 14 *μ*m to 0.73 for 20 *μ*m). Together, these results show that when RhoA can bind to the membrane (cases 4 and 5), YAP/TAZ nuclear translocation increases with cell spreading, whereas the reverse is observed when RhoA is purely cytosolic (case 3).

**Figure 5:**
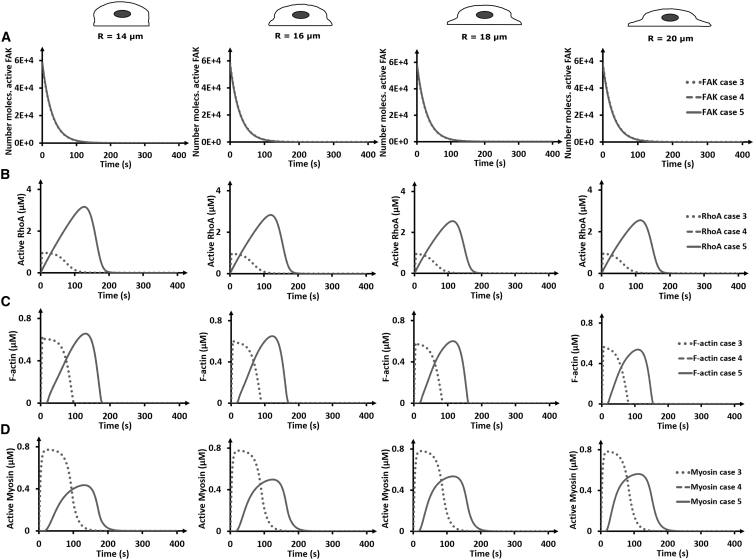
Evolution of active forms of FAK, RhoA, F-actin, and myosin with cell spreading. (*A*) The temporal evolution of the number of molecules (molecs.) of active FAK. Note that all cases overlap. (*B*) The temporal evolution of the concentrations of active RhoA. (*C*) The temporal evolution of the concentrations of F-actin. (*D*) The temporal evolution of the concentrations of myosin. Cases 4 and 5 overlap. The sampled point is located in the membrane in the middle of the cell base for RhoA cases 4 and 5, whereas the point is cytoplasmic for case 3. Myosin and F-actin were sampled in the cytoplasm close to the cell base in all cases. The coordinate values of the sampling points are supplied in the Table S1.

To explain these observations from the mathematical model, we looked at the downstream signaling components (Fig. 5). Although the initial number of molecules of active FAK was set constant across cell spreading states, the evolution of other active, downstream proteins varied with the activation mode (Fig. 5). For case 3, an earlier signaling response is observed than for cases 4 and 5, in which the peak concentrations of active RhoA, F-actin, and active myosin are reached at an earlier time point in contrast to cases 4 and 5. The earlier signaling response of case 3 resulted in smaller peak concentrations of active RhoA but similar peak concentrations of F-actin and higher peak concentrations of active myosin with respect to cases 4 and 5 (Fig. 5, *B*–*D*). Similar to what was observed above for the YTNF, there was a slight decrease in RhoA and F-actin peak concentration with increased cell spreading for case 3 (Tables S2 and S3). Interestingly, whereas in Fig. 4 an increase in YTNF with increased cell spreading was observed for cases 4 and 5, Fig. 5 shows a reduction in peak RhoA concentration with increased cell spreading (see Supporting materials and methods for quantification; Table S2). Similarly, F-actin showed a decrease in concentration with increased cell spreading for cases 4 and 5, whereas myosin showed an increase in concentration with increased cell spreading (Table S4). These results point toward a diversity of signaling concentrations and timing in response to cell spreading and different activation modes.

The above findings illustrate that when both RhoA and FAK are membrane bound, this will lead to downstream signal amplification and increased YAP/TAZ nuclear translocation with increased cell spreading. More specifically, with increased cell spreading, for all cases the local amount of FAK available per unit area to interact with RhoA decreases as the number of active FAK molecules is kept constant across cell sizes and cases. As such, for case 3 in which increased cell spreading does not change the RhoA concentration, this decrease in local FAK concentration results in less RhoA activation and a lower YAP/TAZ fraction with increased cell spreading. However, for cases 4 and 5, this effect can be compensated by the increased surface area. Indeed, the ability for FAK and RhoA to react after RhoA binding to the membrane (thereby increasing its local concentration) is larger because of the increased surface area, resulting in more active RhoA and downstream activation.

### Diffusion coefficients of RhoA and FAK and their binding dynamics influence YAP/TAZ nuclear translocation

**Figure 6:**
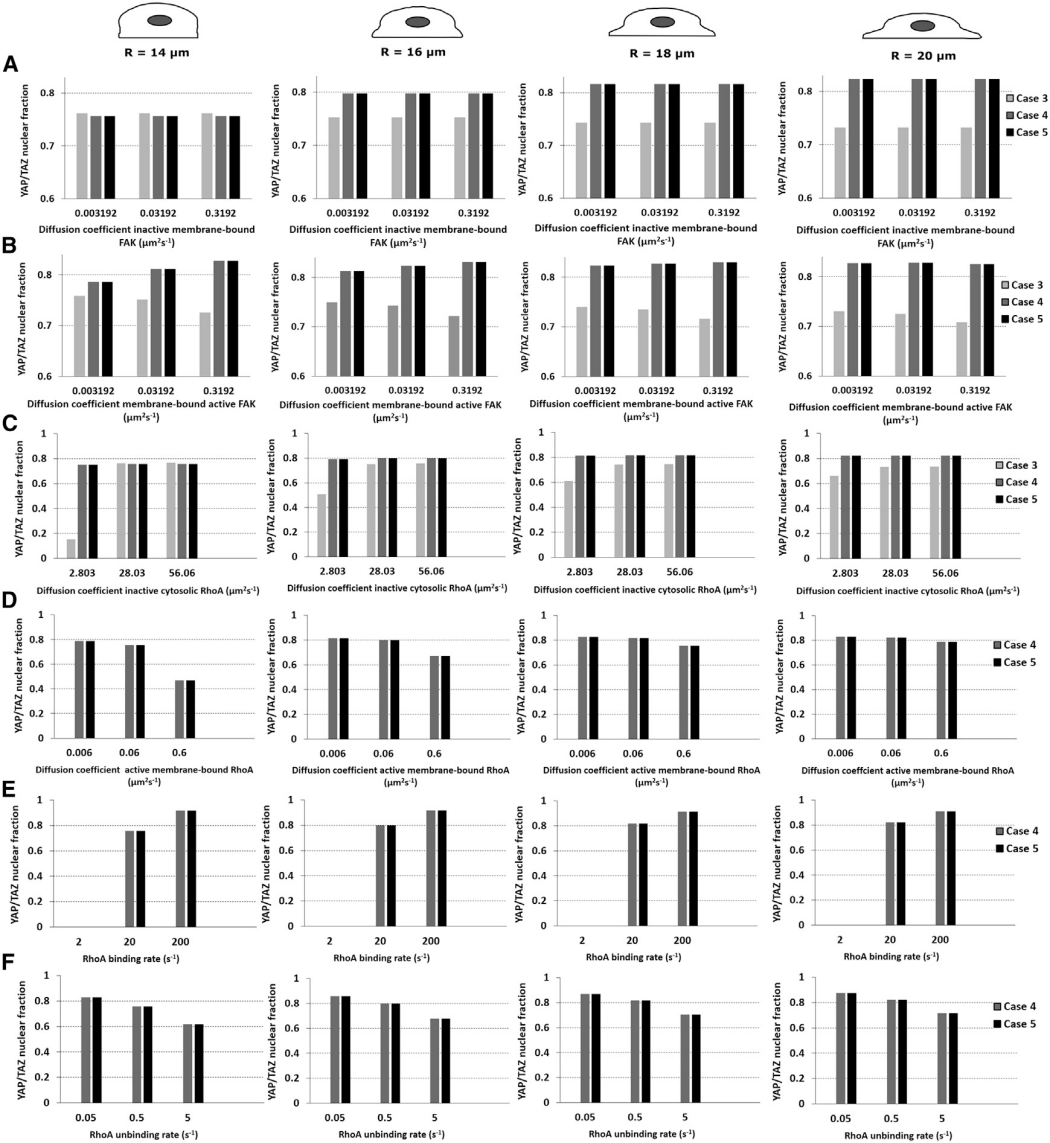
Influence of the diffusion coefficients of FAK and RhoA and binding rates of FAK and RhoA on the YTNF. All values presented here are steady-state values. The panels show the YTNF as a function of the diffusion coefficients of (*A*) membrane-bound inactive FAK, (*B*) membrane-bound active FAK, (*C*) inactive cytosolic RhoA, and (*D*) membrane-bound active RhoA and the binding (*E*) and unbinding (*F*) rates of RhoA, respectively. The middle values represent the standard settings except for FAK (diffusion coefficient is 0 at standard settings).

Diffusion coefficients and binding kinetics are important factors that can affect biological signaling (82,83). Here, we wanted to explore the effect of FAK and RhoA diffusion coefficients on YAP/TAZ nuclear translocation for different cell spreading and activation cases (Figs. 6 and S3).

For case 3, the YTNF decreases with increased diffusion of membrane-bound active FAK (0.75 at 0.003192 *μ*m^2^ s^−1^ vs. 0.72 at 0.3192 *μ*m^2^ s^−1^ for the standard cell size), whereas for cases 4 and 5, the YTNF increases with increased diffusion of membrane-bound active FAK. Interestingly, the influence of the diffusion coefficient of active membrane-bound FAK becomes less important with increased cell spreading (Fig. 6 *A*). This finding is aligned with the above explanation, in which the increased cell spreading results in a lower local FAK concentration but an increased local RhoA concentration (after membrane binding) (for cases 4 and 5). The increased diffusion further increases the surface area in which RhoA and FAK can react, although increased cell spreading counteracts this effect due to the increased diffusion distances. The diffusion coefficients of the inactive forms of FAK have no effect on the YAP/TAZ nuclear response.

For case 3, the YTNF increased with increased diffusion coefficient of the inactive form of RhoA for all cell spreading states up to a saturation level (Fig. 6 *C*), whereas the YTNF was not influenced by the diffusion coefficient of active cytosolic RhoA (Fig. 6 *D*). For cases 4 and 5, the diffusion coefficient of the inactive cytosolic form of RhoA did not influence the YAP/TAZ fraction (Fig. 6 *C*), whereas the YAP/TAZ fraction slightly increased with increased diffusion coefficient of the inactive membrane-bound form of RhoA, although this effect was reduced with increased cell spreading (Fig. S3). Interestingly, the YAP/TAZ fraction decreased with increased diffusion coefficient of the active membrane-bound form of RhoA, although this effect was also reduced with increased cell spreading (Fig. 6 *D*). These results are again aligned with the above explanations that cell spreading results in an increased surface area for RhoA binding and RhoA activation.

Increasing or decreasing the binding rate of inactive cytosolic FAK in case 5 did not affect the YTNF (Fig. S3). Contrarily, an increase in the binding rate of inactive RhoA resulted in an increase in YTNF, whereas the opposite occurred for the unbinding rate of membrane-bound RhoA in cases 4 and 5 (Fig. 6, *E* and *F*). Also here, the influence of the binding rates reduced with increased cell spreading.

Together, these results show that the diffusion coefficients and binding dynamics of RhoA are more important for YAP/TAZ nuclear translocation than those of FAK under these model settings and that cell spreading has a dampening effect.

### FAK unbinding and binding rates influence YAP/TAZ nuclear translocation under sustained activation

**Figure 7:**
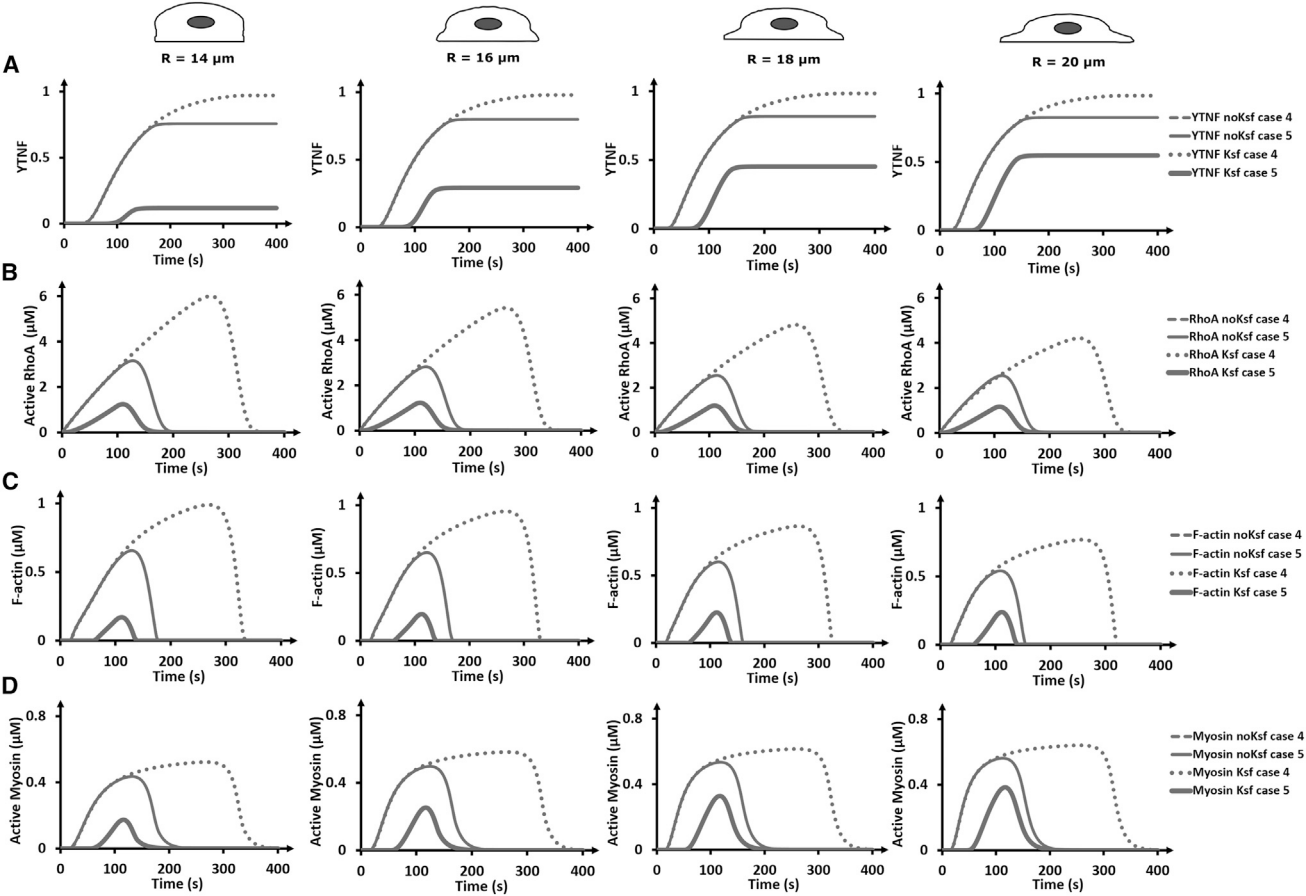
Influence of cell spreading and activation mode on YAP/TAZ nuclear translocation RhoA, F-actin, and myosin evolution for sustained FAK activation (for 100 s via an activation rate *K*_*sf*_). The graphs of sustained FAK activation are compared to those without sustained FAK activation. (*A*) Temporal evolution of the YTNF. The temporal evolution of the concentrations of (*B*) active RhoA, (*C*) F-actin, and (*D*) myosin is given, measured at a point. The graphs of cases 4 and 5 without sustained FAK activation overlap. The sampled point is located in the membrane in the middle of the cell base for RhoA cases 4 and 5, and the point is cytoplasmic. Myosin and F-actin are sampled in the cytoplasm close to middle of the cell base in all cases. The coordinate values of the sampling points are supplied in Table S1.

In the above simulations, no differences were observed between cases 4 and 5 because the simulations were initialized with a particular amount of active FAK, bypassing the (un)binding process that distinguishes these two cases (see Fig. 2). To explore the influence of FAK (un)binding, we modified the initial condition so that membrane-bound inactive FAK is activated at an activation rate *K*_*sf*_ for 100 s (see Eq. S2). Note that because of the influence of cell spreading and FAK (un)binding, this results in different amounts of active FAK and consequently downstream signaling (Figs. 7, S4, and S5). With these settings, all YAP/TAZ translocates to the nucleus for case 4, whereas for case 5, the amount of YAP/TAZ translocation varies between 0.12 and 0.54, pointing toward a high sensitivity with respect to degree of cell spreading (Fig. 7 *A*). The sustained activation signal (compare with and without *K*_*sf*_ in Fig. 7) also results in sustained downstream signaling. It is interesting to note that the signaling starts earlier for case 4 because all the inactive FAK is already at the membrane (and can immediately get activated), whereas for case 5 the inactive cytosolic FAK first needs to bind to the membrane.

**Figure 8:**
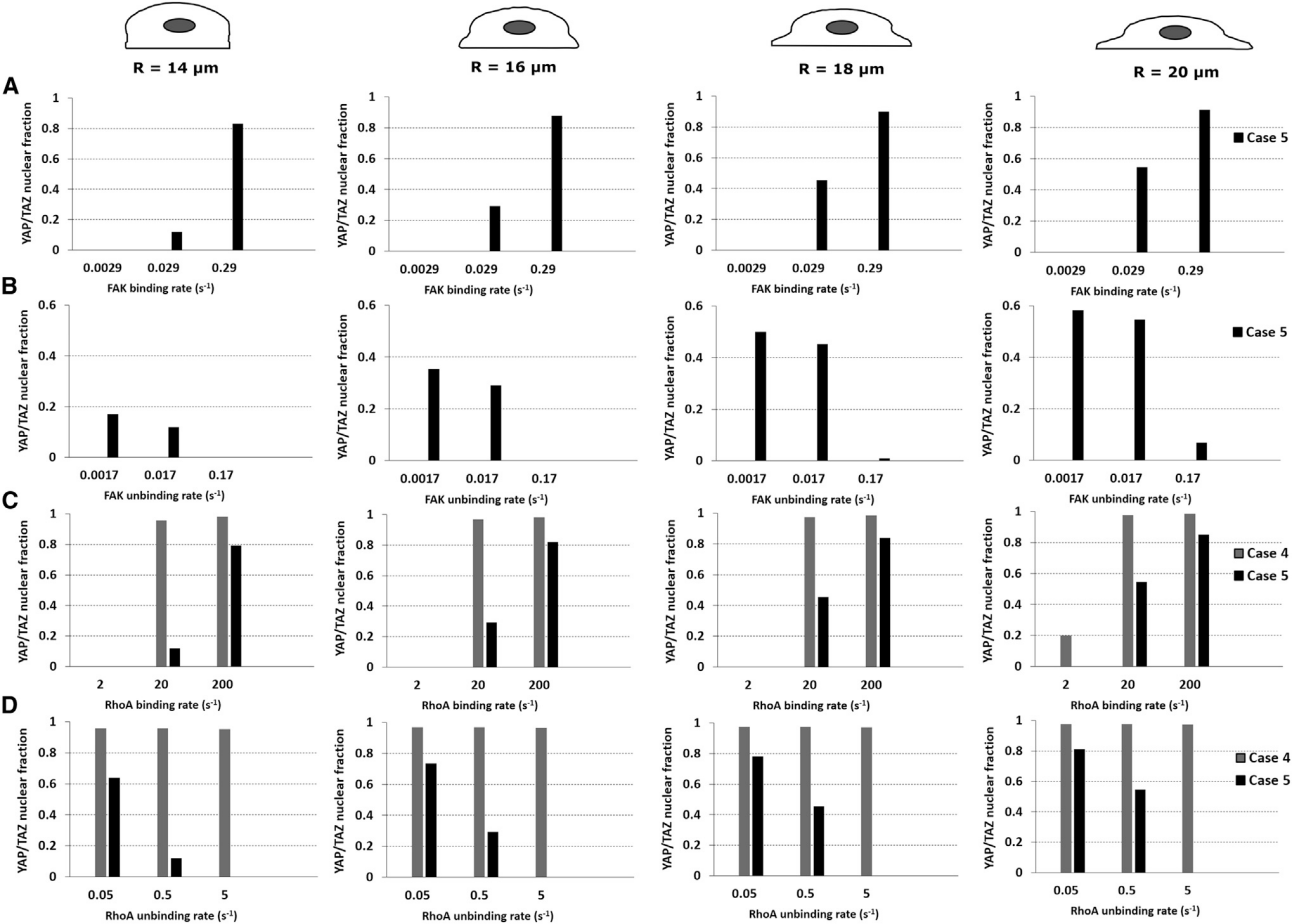
Influence of FAK and RhoA (un)binding on the YTNF. FAK is activated for 100 s via an activation rate (*K*_*sf*_). (*A*) Binding rates of the active cytosolic FAK and (*B*) unbinding rates of the inactive membrane-bound FAK. (*C*) Binding rates of the active cytosolic RhoA and (*D*) unbinding rates of the inactive membrane-bound RhoA. The standard FAK binding and unbinding rates are 0.029 and 0.017 s^−1^, respectively, and the standard RhoA binding and unbinding rates are 20 and 0.5 s^−1^, respectively.

As expected, the steady-state YTNF increases with increasing FAK binding rate (Fig. 8 *A*) and decreases with increased FAK unbinding rate, although this effect is (partially) countered with increased cell spreading (Fig. 8 *B*). Similar observations can be made for RhoA (Fig. 8, *C* and *D*), although case 4 is less sensitive to the RhoA (un)binding rates than case 5. Indeed, in case 4 RhoA is activated earlier (see Fig. 7 *B*) because the inactive FAK is initially membrane bound, whereas in case 5, the inactive FAK first needs to bind to the membrane, get activated, and then, in turn, activate RhoA, resulting in a higher sensitivity to the RhoA (un)binding rates.

## Discussion

Several nonintegral membrane proteins need to bind to the membrane to perform their biological function (17), and it has been shown theoretically (1,20,22,23) and experimentally (21, 22, 23) that cell signaling can be enhanced when these proteins interact with the plasma membrane. Particularly, the formation of membrane receptor clusters and rafts by membrane-bound molecules helps concentrate the signal to specific areas of the cell membrane and amplify signals from the membrane (19, 20, 21, 22, 23, 24). In this in silico study, we have shown that similar mechanisms are at play for the YAP/TAZ signaling pathway. More specifically, our results indicated that YAP/TAZ only translocated to the nucleus when the input signal FAK is membrane bound (cases 3–5). When FAK is activated in the cytoplasm, independent of whether RhoA is membrane bound or cytoplasmic, there was no YAP/TAZ nuclear translocation. Using a rigorous sensitivity analysis, we found that the membrane diffusion of the active forms of FAK and RhoA and their membrane binding dynamics were important regulators of YAP/TAZ nuclear translocation. Moreover, building on the work of others (27,28), we have confirmed that similar to 2D (3,9,27,84), YAP/TAZ nuclear translocation increases with cell spreading in three dimensions under particular conditions (28,76,85,86).

To study the role of membrane signal amplification in YAP/TAZ signaling, we developed a computational model for five types of FAK activation by distinguishing membrane-unbound (cases 1 and 2) and membrane-bound (cases 3–5) FAK activation. We observed that membrane localization of FAK contributes to a robust YAP/TAZ signal downstream. These results indicate that the membrane recruitment of FAK and anchoring to the membrane is important for YAP/TAZ signal amplification, similar to other signaling pathways (19,21,23, 24, 25). To explain the signal amplification through membrane localization, one needs to compare the dimensionality of the membrane (2D) with the one of the cytoplasm (3D). The reduced dimensionality of the membrane implies that the same number of active FAK molecules at the membrane (cases 3–5; Eq. 5) would translate into a higher initial active FAK concentration at the membrane compared to the cytosolic cases (cases 1 and 2; Eq. 5). Similarly, Schmick et al. (87) demonstrated that by considering the cytosol as a sphere and the plasma membrane as a shell around the cytosol, the concentration of a signaling effector initially diffusing in the cytosol would increase by ∼1000-fold if recruited and confined to diffusion on the membrane. Moreover, this type of increase in signaling molecule concentration at the membrane trumps the effects of reduced diffusion at the membrane (87). Interestingly, membrane localization of only RhoA (case 2) does not lead to a YAP/TAZ response. Indeed, for our cell size and initial number of FAK molecules, when RhoA and FAK do not colocalize at the membrane through (un)binding, it is impossible to trigger strong enough signals downstream to exceed the ROCK and mDia thresholds. Furthermore, we see an early attainment of peak YAP/TAZ concentration in case 3 compared to cases 4 and 5, which is consistent with the notion that cell signaling efficiency is dependent on the spatiotemporal organization of the signaling components (25,75,88). Therefore, not only membrane recruitment but also the particular signaling entity (in the specific pathway) being recruited are important.

Our in silico results underscore the need for taking into consideration membrane binding and unbinding dynamics of FAK and RhoA for YAP/TAZ signaling. It has been pointed out that the biological activity of several nonintegral membrane proteins is dependent on their membrane anchoring and thus their membrane binding and unbinding dynamics (17,18). However, despite the pivotal role played by membrane interactions in the activity of these proteins, it has been very difficult to quantify their membrane binding and unbinding rates experimentally (17). We have shown theoretically that YAP/TAZ nuclear translocation can be modulated by the membrane binding kinetics of RhoA and FAK. We observed an increase in the YTNF for higher inactive cytosolic RhoA binding rates. Indeed, the higher the binding rate, the more transfer of RhoA molecules to the membrane and the higher the chances of interaction with active FAK within the same membrane compartment. To investigate the effect of FAK binding and unbinding dynamics, we introduced a FAK activation rate instead of a fixed amount of initial active FAK. Interestingly, a high inactive cytosolic FAK binding rate increased the amount of inactive membrane-bound FAK available for activation at the membrane, thus leading to increased downstream activation and resulting in higher YTNF.

Our results agree with observations reported in literature (28,76,85,86) that the YTNF increases with cell spreading when RhoA is membrane bound. For example, in their study on the control focal adhesion by YAP signaling, Nardone et al. (89) showed that the YAP/TAZ nuclear signal increased with cell spreading, consistent with our results for cases 4 and 5. In their modeling work on the role of substrate stiffness, substrate dimensionality, and cell shape on YAP/TAZ signaling, Scott et al. (28) were able to predict that for a constant cell volume, YAP/TAZ would increase with substrate activation area in three dimensions (referred to as 2.XD) at medium (5.7 kPa) and high (7 GPa) stiffnesses. Their 2.XD corresponds to a 3D cell in which activation of FAK (and RhoA) is restricted to the base, similar to our simulation setup. Interestingly, although their approach is congruent with the need for RhoA to be membrane bound to obtain an increase in YAP/TAZ output with cell spreading (cases 4 and 5), their spatial model results do predict YAP/TAZ nuclear translocation when FAK is cytosolic and activated in the cytosol (28), in contrast to our results, in which a predetermined amount of active FAK did not result in YAP/TAZ translocation for cases 1 and 2. Similarly, in their 1D exploration, Sun et al. (27) were able to show that YAP/TAZ nuclear translocation does occur in a 1D paradigm and varies with substrate stiffness. Here, to study the influence of membrane localization on YAP/TAZ nuclear translocation, we triggered the YAP/TAZ signaling dynamics by defining a predetermined amount of active FAK, which was kept constant across all cases. This predetermined amount did not result in YAP/TAZ translocation for cases 1 and 2, although we show that higher amounts of initial active FAK do result in YAP/TAZ nuclear translocation for cases 1 and 2, similar to previous nonspatial (27) and spatial (28) models (see Fig. S6). More specifically, for their 2.XD setup with similar activation areas, Scott et al. (28) obtained a lower YTNF (i.e., 0.29) compared to the ones obtained in this study for membrane-bound cases (i.e., 0.8). This difference in YTNF may be explained by the fact the Scott et al. (28) models YAP/TAZ nuclear export, whereas we set this to zero. Moreover, Scott et al. (28) include a continuous FAK activation, whereas this work starts from a fixed amount of initial active FAK, which may explain the need for higher amounts of initial active FAK for cases 1 and 2 in this modeling framework. In summary, we highlight that FAK membrane binding is not essential to achieve YAP/TAZ nuclear translocation in our modeling framework but results in a higher and more robust YAP/TAZ response.

The modeling work of Sun et al. (27) and Scott et al. (28) has also described and studied FAK activation as a function of substrate stiffness. As this was not the main goal of this work, we only performed a small auxiliary experiment (see Fig. S7), in which we alter the stiffness for sustained FAK activation for case 5 on a standard cell of radius 16 *μ*m. The results show that stiffness sensing occurs in our model for 0–50 kPa. For similar FAK activation areas (402 *μ*m^2^ vs. 415 *μ*m^2^ in (28)), our stiffness sensing range (0–50 kPa) is lower than the one of Scott et al. (28) (0–100 kPa) and higher than in the 1D model of Sun et al. (27) (0–20 kPa). The differences in stiffness sensing ranges may arise from the fact that Scott et al. (28) also define a cytosolic stiffness, which is linked to the F-actin concentration, and relate it to the nuclear mechanics (lamin A activation).

Importantly, similar to our computational predictions, experimental literature as shown that the membrane localization of FAK or RhoA influences downstream YAP/TAZ signaling. For example, small molecules such as dasatinib, pazopanib, and lovastatin, which inhibit Rho GTPase prenylation and thereby prevent membrane localization, were found to reduce the nuclear localization of YAP (54,55,90). Oku et al. (55) have shown that 300 nM of dasatinib reduced the percentage of cells expressing nuclear YAP/TAZ from ∼80 to 5%. This corresponds to our predictions for case 1, in which there is no YAP/TAZ nuclear translocation. Interestingly, for case 3, in which RhoA is also cytoplasmic but FAK is membrane bound, the computational model does predict YAP/TAZ nuclear translocation. This difference may indicate that in the in vitro experiments mechanisms other than spatial location play a role in inhibiting YAP/TAZ translocation. In particular, by preventing membrane localization, the small molecules also reduce Rho GTPase activation, an effect we did not include in the model because we focused on the influence of localization only. Alternative splicing of FAK was shown to result in reduced focal adhesion formation, increased cytoplasmic localization of FAK, and reduced nuclear YAP (56,57), corresponding to the computational predictions in which the YAP/TAZ nuclear translocation is absent for cases 1 and 2 for the standard FAK activation scheme.

Next to membrane binding and unbinding dynamics, diffusion also plays an important role in cell signaling (1,2,25,91,92). Although this presents an avenue for experimental investigation, we note that the diffusion of active forms of FAK and RhoA could potentially modulate the YAP/TAZ nuclear output. We observed that an increase in the diffusion rate of active membrane-bound FAK resulted in an increase in the YTNF in cases 4 and 5 and a decrease in the YTNF in case 3. Interestingly, the increase in YTNF in cases 4 and 5 was attenuated with increased cell spreading. These results can be explained as follows: an increase in the diffusion coefficient of active FAK increases the speed of encounter of active FAK with inactive membrane-bound RhoA within the membrane, thus leading to more RhoA molecules being activated in cases 4 and 5. However, with increased cell spreading leading to larger distances to be traveled by diffusion, this effect is reduced. For case 3, an increasing diffusion coefficient of active FAK reduces the (high) local FAK concentrations at the base of the cell, resulting in less RhoA activation (in the cytosolic area near the base of the cell). Similar to our findings of case 5, Scott et al. (28), who model cytosolic inactive FAK and activation of FAK at the plasma membrane, report an increase in YAP/TAZ fraction with increased diffusion coefficients. They also report that this effect is reduced for larger activation areas (e.g., with increased spreading), in agreement with our findings.

The results of this study, which summarize the role of membrane localization and binding and unbinding dynamics, diffusion, and cell spreading on YAP/TAZ nuclear localization, should be interpreted in the light of the following assumptions and limitations. Firstly, we do not account for the discrete nature of focal adhesions at the membrane, but rather assume that focal adhesion molecule activation and exchange happens in a continuous region in contact with the substrate. Furthermore, we assume that the signaling cascade is solely dependent on initial FAK activation, ignoring any signaling cross talk. Secondly, considering the short timescales that we model, we assume a constant amount of protein, thus ignoring potential production and degradation processes. Thirdly, we model a fixed nuclear volume and shape and a fixed distance of the nucleus from the center of the base of the cell, all of which are able to undergo dynamic changes with cell spreading. Finally, the current values of membrane exchange rates, especially for FAK, are estimated within ranges of scarcely available data. Experimental work needs to be done to obtain more accurate rate values within various cell spreading and environmental stiffness contexts, by using approaches such a fluorescence recovery after photobleaching.

In summary, in this study we investigated the effect of FAK and RhoA membrane binding on YAP/TAZ signaling. We showed that FAK membrane binding can modulate and amplify the YAP/TAZ nuclear response. Moreover, we predicted an increase of YTNF with increased cell spreading, but only when FAK and RhoA are membrane bound. Future work should focus on the experimental verification of our predictions, namely on monitoring membrane interaction of FAK and RhoA and their effect on YAP/TAZ nuclear signal enhancement in relation to cell shape and dimensionality. By investigating the influence of membrane activation on downstream signaling, a motif common to many signaling pathways, this study contributed to an improved understanding of the design principles of signaling networks.

## Author contributions

K.S.E. designed and performed research, analyzed data, and wrote manuscript. R.C. designed and supervised research and gave feedback on manuscript. K.S. designed and supervised research and gave feedback on manuscript. J.d.B. supervised research and gave feedback on manuscript. A.C. designed and supervised research, gave feedback on manuscript, edited manuscript, and acquired financial support.

## Supporting material

Document S1. Supporting materials and methods, Figs. S1–S7, and Tables S1–S4

Document S2. Article plus supporting material
