# Supplementary material for: Exploring the influence of cytosolic and membrane FAK activation on YAP/TAZ nuclear translocation
Source: Biophys J. 2021 Sep 10;120(20):4360–77. doi: 10.1016/j.bpj.2021.09.009 (PMC8553670; doi:10.1016/j.bpj.2021.09.009)
Supplement: Document S1. Supporting materials and methods, Figs. S1–S7, and Tables S1–S4 [file mmc1.pdf]

Biophysical Journal, Volume *120*

Supplemental information

Exploring the influence of cytosolic and membrane FAK activation on YAP/TAZ nuclear translocation

Kerbaï Saïd Eroumé, Rachel Cavill, Katerina Staňková, Jan de Boer, and Aurélie Carlier

Supplementary

Model use and access

The models for a standard cell of base radius 16 µm, can be accessed on the VCell public model repository https://vcell.org/vcell-published-models. The names of the models are as follows, for models without sustained FAK activation (for 100s):

Kerbai_Biophys.J_2021_YAPTAZ_noKsf_case1,
Kerbai_Biophys.J_2021_YAPTAZ_noKsf_case2,
Kerbai_Biophys.J_2021_YAPTAZ_noKsf_case3,
Kerbai_Biophys.J_2021_YAPTAZ_noKsf_case4,
Kerbai_Biophys.J_2021_YAPTAZ_noKsf_case5.

For models with sustained FAK activation (for 100s):

Kerbai_Biophys.J_2021_YAPTAZ_Ksf_case3,
Kerbai_Biophys.J_2021_YAPTAZ_Ksf_case4,
Kerbai_Biophys.J_2021_YAPTAZ_Ksf_case5.

Details on running a model in VCell can be found in the quick start guide on the VCell website, https://vcell.org/support.

Stokes-Einstein derivation of diffusion coefficients

By assuming molecules to be spherical with radius r, we can use the formula proposed by stokes and Einstein to estimate the diffusion coefficients (D) of molecules [1].

$$D = \frac{K * T}{6 * \pi * \eta * r} \qquad [1]$$

Where K = Boltzmann constant, T is the temperature = 300 K (room temperature), η = viscosity of medium in which the molecules are found, and r the equivalent spherical molecular radius.

By using the relationship of the viscosity of cytosol with respect to water $\eta_{cytosol} = 11 * \eta_{water}$

[Kalwarczyk et al. 2011] we can calculate the diffusion coefficient of each molecule in the YAP/TAZ signaling pathway. The equivalent radii were determined using the radius calculator found at http://www.calctool.org/CALC/prof/bio/protein_size. This tool determines the equivalent radius based on the number of amino acid residues of each molecule. We obtained the number of residues by using the protein data bank.

Typical cell of radius R = 16 µm

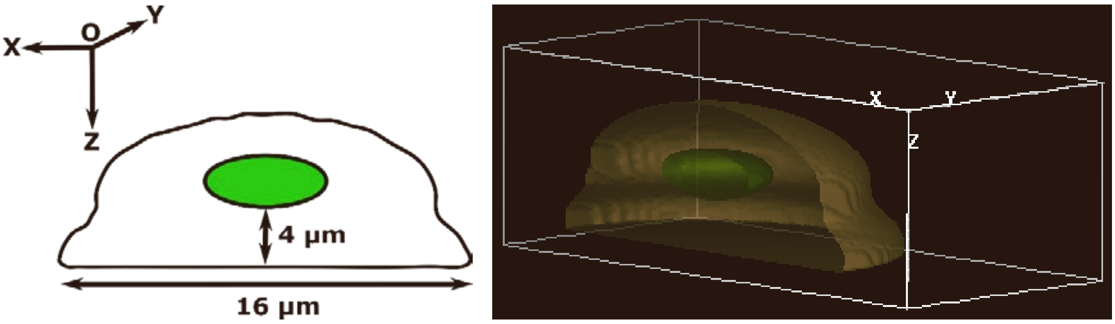

Figure S1: Typical cell or radius R = 16 µm showing the nucleus in green. 2D cross-section on the left and a 3D view on the right. The origin is taken at coordinate point O (-23 µm, 0 µm, -14 µm).

Description of sampling points:

The following coordinate points were used to obtain the concentrations in the membrane, cytoplasm and in the nucleus.

Membrane (0.29 µm, 0 µm, -0.33 µm), Cytoplasm (0.29 µm, 0 µm, -0.66 µm), Nucleus (0.29 µm, 0 µm, -6 µm)

Table S1: Species and corresponding sampling points for their concentrations (µM) as shown in Figures 3, 5 and 7. M, C and N represent Membrane, Cytoplasm and Nucleus respectively. Here YAP/TAZ refers to nuclear YAP/TAZ.

Species	Case 1	Case 2	Case 3	Case 4	Case 5
Active FAK	C (0.29, 0 , -0.66)	C (0.29, 0 , -0.66)	M(0.29, 0 , -0.33)	M(0.29, 0 , -0.33)	M(0.29, 0 , -0.33)
Active RhoA	C (0.29, 0 , -0.66)	M(0.29, 0 , -0.33)	C (0.29, 0 , -0.66)	M(0.29, 0 , -0.33)	M(0.29, 0 , -0.33)
F-actin	C (0.29, 0 , -0.66)	C (0.29, 0 , -0.66)	C (0.29, 0 , -0.66)	C (0.29, 0 , -0.66)	C (0.29, 0 , -0.66)
Myosin	C (0.29, 0 , -0.66)	C (0.29, 0 , -0.66)	C (0.29, 0 , -0.66)	C (0.29, 0 , -0.66)	C (0.29, 0 , -0.66)
YAP/TAZ	N (0.29, 0 , -6)	N (0.29, 0 , -6)	N (0.29, 0 , -6)	N (0.29, 0 , -6)	N (0.29, 0 , -6)

Equation of FAK activation through stiffness and activation rate

This equation holds for cases 3, 4 and 5 on the plasma membrane.

	FAK	
	$$\frac{\partial FAK}{\partial t} = K_{sf}.\left(\frac{(LD * E_{mol})^2}{(C^2 + (LD * E_{mol}))^2}\right).FAK_{mi} - K_{df}.FAK + D_{FAK}.\nabla^2 FAK$$	[2]

At the plasma membrane we have a no flux boundary condition for FAK and FAK_{mi} in cases 3 and 4, while in case 5 we have a no flux boundary condition for FAK and the boundary condition for FAK_{ci} and FAK_{mi} (see Eq. 18 in main manuscript) is such that the (un)binding events are in balance with the diffusive flux:

	$- D_{FAK_{ci}} \cdot\ e_n \cdot \nabla_V FAK_{ci} = N \cdot (KF_{on} \cdot FAK_{ci} - KF_{off} \cdot FAK_{mi})$	[3]

Table S2: Quantifications of peak RhoA concentrations (µM), without sustained FAK activation for the standard parameter settings (see Table 1), as shown in Figure 5.

	R = 14 µm	R = 16 µm	R = 18 µm	R = 20 µm
Case 3	0.96	0.95	0.95	0.94
Case 4	3.16	2.84	2.55	2.29
Case 5	3.16	2.84	2.55	2.29

Table S3: Quantifications of peak F-actin concentrations (µM), without sustained FAK activation for the standard parameter settings (see Table 1) as shown in Figure 5.

	R = 14 µm	R = 16 µm	R = 18 µm	R = 20 µm
Case 3	0.63	0.60	0.57	0.56
Case 4	0.66	0.65	0.60	0.54
Case 5	0.66	0.65	0.60	0.54

Table S4: Quantifications of peak myosin concentrations (µM), without sustained FAK activation for the standard parameter settings (see Table 1) as shown in Figure 5.

	R = 14 µm	R = 16 µm	R = 18 µm	R = 20 µm
Case 3	0.77	0.78	0.78	0.78
Case 4	0.44	0.50	0.54	0.56
Case 5	0.44	0.50	0.54	0.56

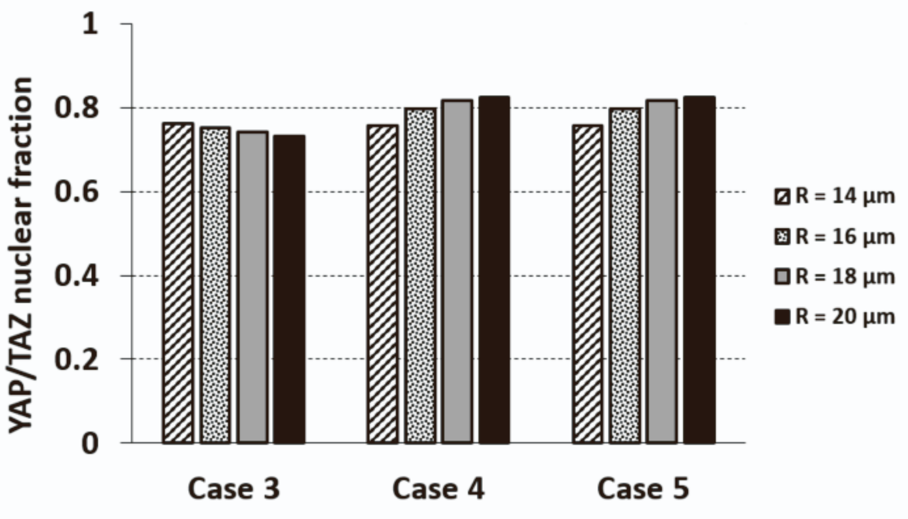

Figure S2. Influence of cell spreading and activation mode on YAP/TAZ nuclear translocation. Evolution of the steady state YAP/TAZ nuclear fraction with cell spread for a fixed amount of initial active FAK (also see Figure 4).

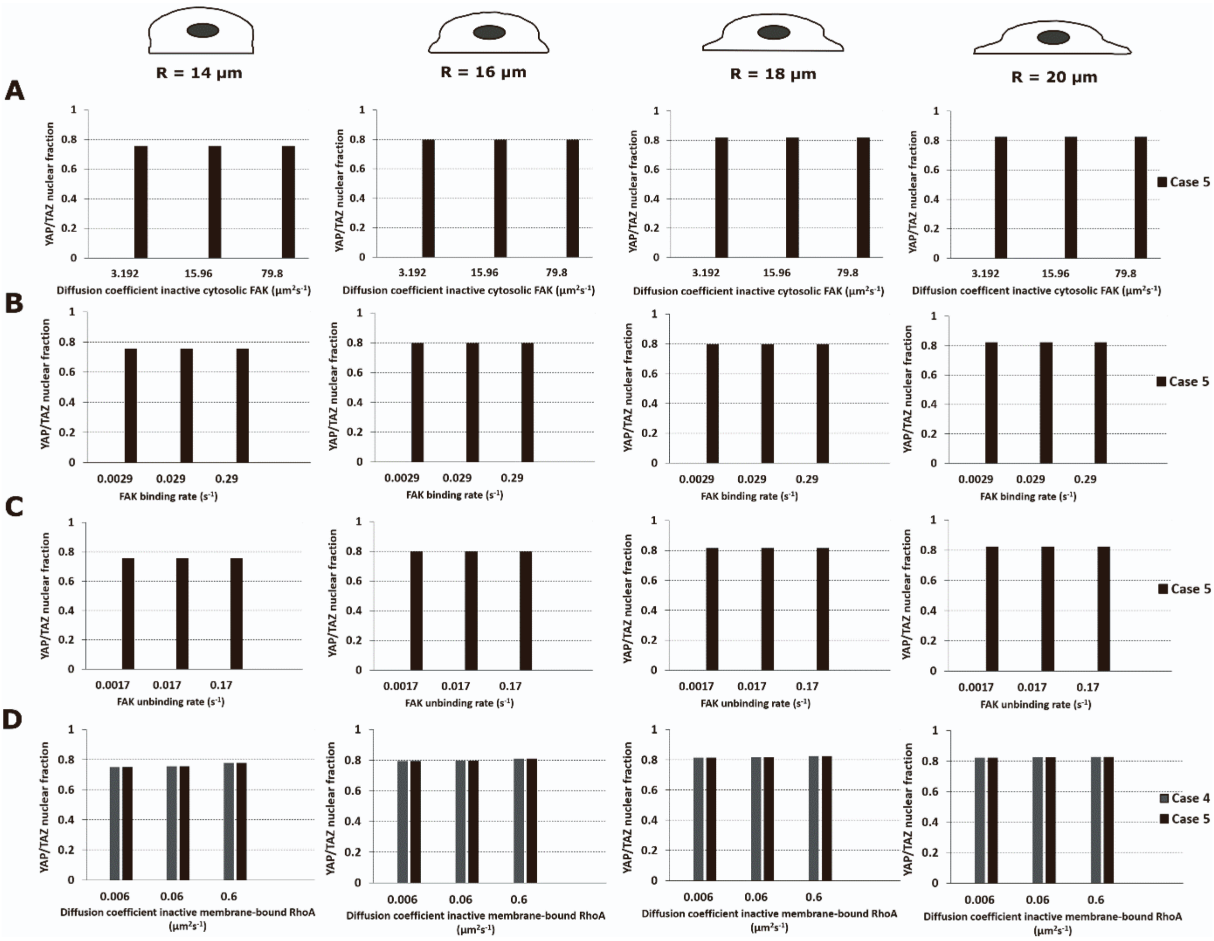

Fig. S3. Influence of diffusion of FAK, RhoA, and binding rates of active FAK on the YAP/TAZ nuclear fraction for the model with a fixed initial amount of active FAK. The middle values represent the standard settings except for FAK (diffusion coefficient is 0 at standard settings).

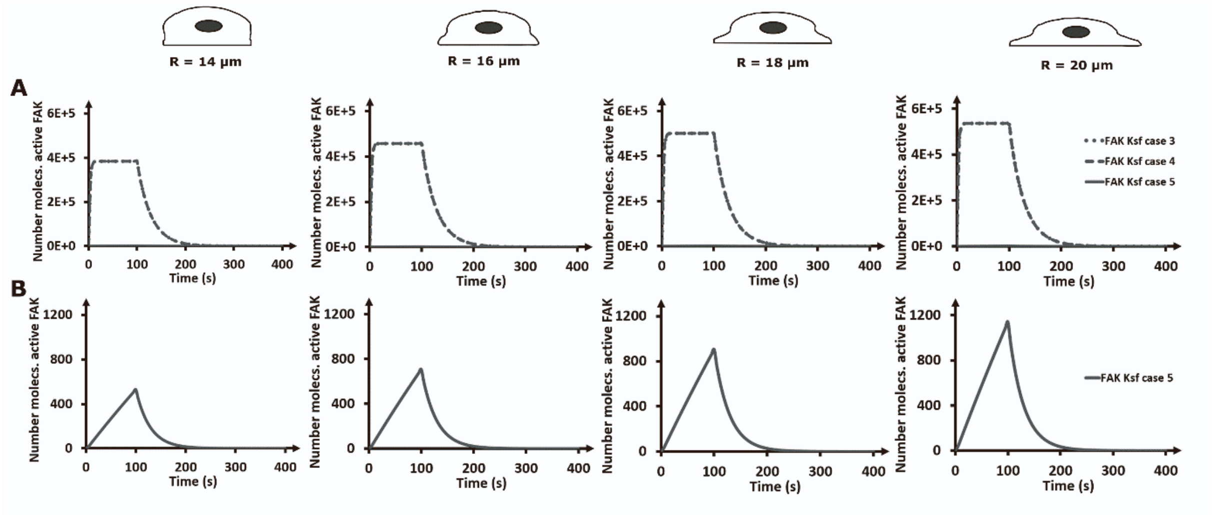

Fig S4: Evolution of number of molecules of active FAK with cell spreading; sustained activation through an activation rate Ksf for 100s. A) Case 3, 4 and 5 combined. Case 5 shows very low FAK levels compared to other cases hence its evolution is shown in B).

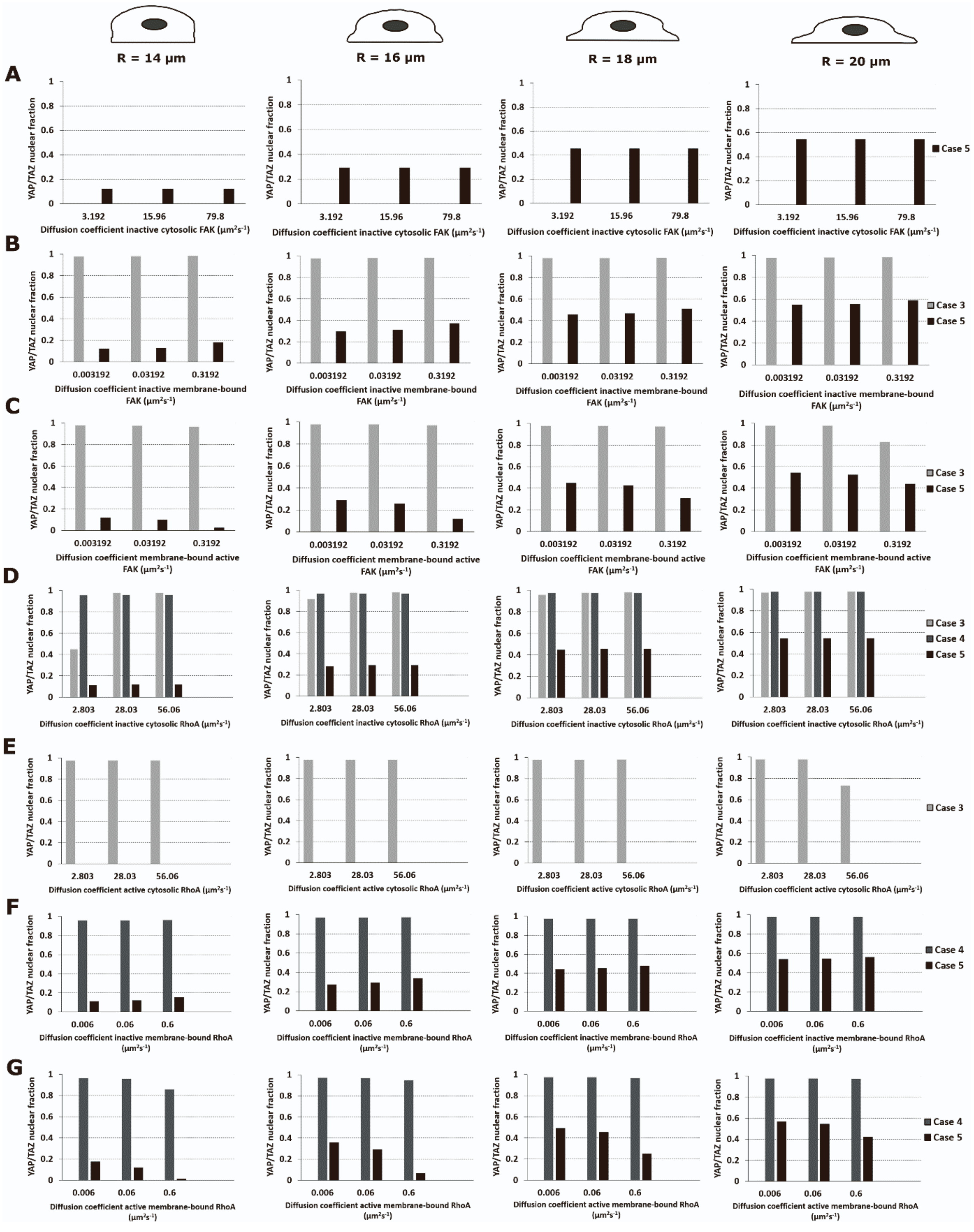

Fig. S5. Influence of diffusion of FAK, RhoA, and binding rates of active FAK on the YAP/TAZ nuclear fraction for model with sustained FAK activation (via rate Ksf). All values presented here are steady-state values. The panels show the YAP/TAZ nuclear fraction as a function of the diffusion coefficients of A) inactive cytosolic FAK B) membrane-bound inactive FAK C) membrane-bound active FAK D) inactive cytosolic RhoA (E) active cytosolic RhoA (F) inactive membrane-bound RhoA G) active membrane-bound RhoA. The middle values represent the standard settings except for FAK (diffusion coefficient is 0 at standard settings).

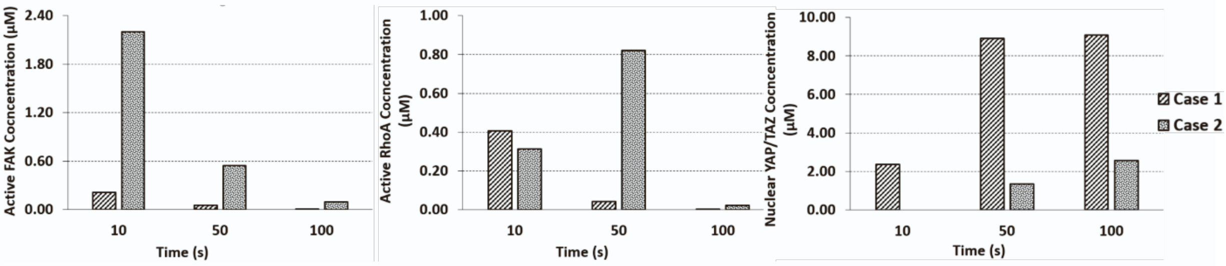

Figure S6. Overview of spatiotemporal predictions of the YAP/TAZ model for cases 1 and 2 for a fixed amount of initial active FAK. Temporal evolution of concentrations of active FAK, RhoA and nuclear YAP/TAZ for realistic cells with base radii 16 µm for a higher initial concentration of FAK in case 1 (0.3 µM) and case 2 (3.12 µM at the strip of height 0.5 µm at the base of the cell). The number of initial active FAK molecules are kept constant across the cases. The sampled point for FAK in both cases and RhoA in case 1, is located in the cytoplasm in the middle of the cell base while it is located in the cell membrane, in the middle of the cell base for RhoA in case 2.

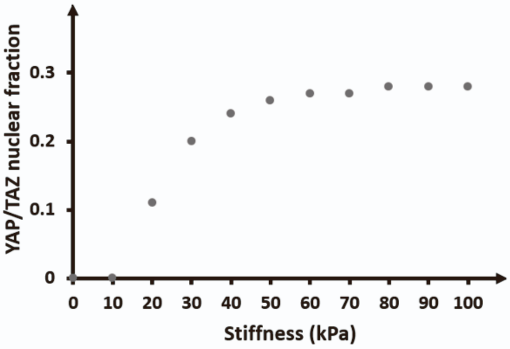

Figure S7. YAP/TAZ nuclear fraction as a function of substrate stiffness. Evolution of the steady state YAP/TAZ nuclear fraction with stiffness for a standard cell (radius 16 µm) for case 5 and a sustained initial FAK activation for 100s via an activation rate (K_{sf}).
